# The dynamic equilibrium between the protective and toxic effects of matrine in the development of liver injury: a systematic review and meta-analysis

**DOI:** 10.3389/fphar.2024.1315584

**Published:** 2024-01-29

**Authors:** Weiyi Feng, Te-chan Kao, Jiajie Jiang, Xinyu Zeng, Shuang Chen, Jinhao Zeng, Yu Chen, Xiao Ma

**Affiliations:** ^1^ TCM Regulating Metabolic Diseases Key Laboratory of Sichuan Province, Hospital of Chengdu University of Traditional Chinese Medicine, Chengdu, China; ^2^ State Key Laboratory of Southwestern Chinese Medicine Resources, Chengdu University of Traditional Chinese Medicine, Chengdu, China; ^3^ School of Clinical Medicine, Chengdu University of Traditional Chinese Medicine, Chengdu, China; ^4^ School of Pharmacy, Chengdu University of Traditional Chinese Medicine, Chengdu, China; ^5^ Hospital of Chengdu University of Traditional Chinese Medicine, Chengdu, China

**Keywords:** matrine, hepatotoxicity, hepatoprotection, liver injury, meta-analysis

## Abstract

**Background:** Matrine, an alkaloid derived from the dried roots of *Sophora flavescens* Aiton, has been utilized for the treatment of liver diseases, but its potential hepatotoxicity raises concerns. However, the precise condition and mechanism of action of matrine on the liver remain inconclusive. Therefore, the objective of this systematic review and meta-analysis is to comprehensively evaluate both the hepatoprotective and hepatotoxic effects of matrine and provide therapeutic guidance based on the findings.

**Methods:** The meta-analysis systematically searched relevant preclinical literature up to May 2023 from eight databases, including PubMed, Web of Science, Cochrane Library, Embase, China National Knowledge Infrastructure, WanFang Med Online, China Science and Technology Journal Database, and China Biomedical Literature Service System. The CAMARADES system assessed the quality and bias of the evidence. Statistical analysis was conducted using STATA, which included the use of 3D maps and radar charts to display the effects of matrine dosage and frequency on hepatoprotection and hepatotoxicity.

**Results:** After a thorough screening, 24 studies involving 657 rodents were selected for inclusion. The results demonstrate that matrine has bidirectional effects on ALT and AST levels, and it also regulates SOD, MDA, serum TG, serum TC, IL-6, TNF-α, and CAT levels. Based on our comprehensive three-dimensional analysis, the optimal bidirectional effective dosage of matrine ranges from 10 to 69.1 mg/kg. However, at a dose of 20–30 mg/kg/d for 0.02–0.86 weeks, it demonstrated high liver protection and low toxicity. The molecular docking analysis revealed the interaction between MT and SERCA as well as SREBP-SCAP complexes. Matrine could alter Ca^2+^ homeostasis in liver injury via multiple pathways, including the SREBP1c/SCAP, Notch/RBP-J/HES1, IκK/NF-κB, and Cul3/Rbx1/Keap1/Nrf2.

**Conclusion:** Matrine has bidirectional effects on the liver at doses ranging from 10 to 69.1 mg/kg by influencing Ca^2+^ homeostasis in the cytoplasm, endoplasmic reticulum, Golgi apparatus, and mitochondria.

**Systematic review registration:** https://inplasy.com/, identifier INPLASY202340114

## Graphical Abstract

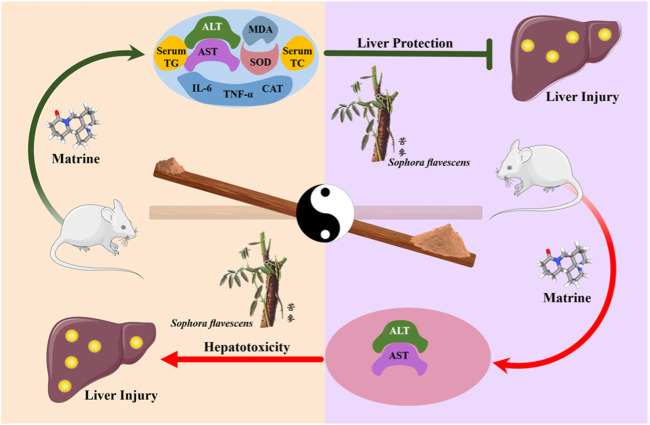

## 1 Introduction

Liver injury (LI) is a prevalent liver disease and a significant global health concern due to its high mortality rates. The European Association for the Study of the Liver (EASL) has reported that liver disease ranks 11th as the leading cause of death worldwide, accounting for 4% of all deaths. In 2023, Devarbhavi et al. estimated over two million fatalities annually due to liver disease (Devarbhavi et al., 2023). As a preliminary stage of liver disease, LI can be caused by various factors such as alcohol, infection, immunity, and drug-induced toxicity (Younossi et al., 2023). The severity of LI can range from mild inflammation to more severe conditions like liver cirrhosis, liver failure and even death. Symptoms of LI consist of abnormalities in liver function test abnormalities, fever, nausea, vomiting, jaundice, and right epigastric pain (Knight, 2005). Hepatocellular damage, fibrosis, and inflammatory infiltration are key pathological features of LI. Anti-viral drugs, liver protective agents, and immunosuppressive drugs are the mainstream drugs used to treat LI. While corticosteroids, pioglitazone, cholestyramine, and other medications are commonly prescribed to treat different types of LI (Devarbhavi et al., 2023), their hepatotoxicity can lead to drug-induced liver injury (DILI) in clinical settings. Therefore, exploring more effective and safer alternatives for LI is necessary.

The dried roots of *Sophora flavescens* Aiton (well-documented in The Plant List www.theplantlist.org), a Traditional Chinese Medicine (TCM) herb, commonly known as KuShen, was initially discovered for its therapeutic properties in the ancient text *Shen Nong Ben Cao Jing*. For thousands of years, the dried roots of *S. flavescens* Aiton has been widely used to treat various digestive disorders, such as dysentery, bloody stools, jaundice, and especially liver diseases (Chinese Pharmacopoeia Commission, 2020). Kushen Decoction and Longdan Kushen Decoction are the representative TCM prescriptions that incorporate the dried roots of *S. flavescens* Aiton for treating liver disease. Contemporary pharmacological studies have revealed that extracts of the dried roots of *S. flavescens* Aiton have hepatoprotective effects due to their anti-inflammatory and antiviral properties (He et al., 2015).

**FIGURE 1:**
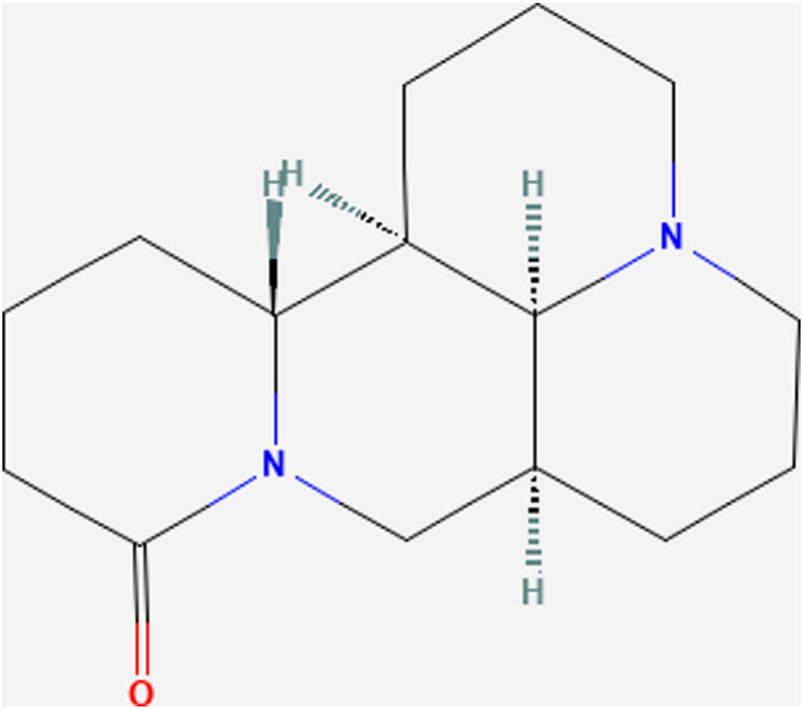
Chemical structure of Matrine. (PubChem Identifier: CID 91466, URL: https://pubchem.ncbi.nlm.nih.gov/compound/91466#section=2D-Structure).

Matrine ((1R,2R,9S,17S)-7,13-diazatetracyclo [7.7.1.02,7.013,17]heptadecan-6-one; C_15_H_24_N_2_O; MW = 248.36) is an alkaloid extracted from the dried roots of *S. flavescens* Aiton and can be dissolved in various solvents such as water, ethanol, benzene, etc .,(Sun et al., 2022). As an active ingredient of the dried roots of *S. flavescens* Aiton, the total content of matrine and oxymatrine should not be less than 1.2% according to the Pharmacopoeia of the People’s Republic of China (Chinese Pharmacopoeia Commission, 2020). Numerous studies have shown that MT has anti-inflammatory, anti-viral, anti-tumor, and immune-suppressive abilities (Zhou et al., 2014; Wang et al., 2018; Peng et al., 2020; Chu et al., 2021; Jing et al., 2021). MT has been reported to regulate liver protective function, hepatic regeneration, and alleviate LI through several signaling pathways, such as TGF-β/Smad, NF-κB, Wnt/β-catenin, Notch/Jagged1/recombination signal binding protein for immunoglobulin kappa J (RBP-Jκ, RBP-J)/hairy and enhancer of split-1 (HES1) (Yu H. B. et al., 2011; Yu et al., 2014; Yang et al., 2016; Yin et al., 2018). Due to its extensive pharmacological effects, MT is often used as an injection in clinical practice for hepatitis B, tumors, and immune diseases (Liu and Zhang, 2021). In the clinical pharmacokinetic study of MT, serum MT concentrations ranged from 1 to 6 ug/mL after a large-dose intravenous infusion (6 mg/kg) (Zhang et al., 2009). In rats, the maximum blood concentration of MT was found to reach 2,412 ± 362 ng/mL and 94.6 ± 38.6 ng/mL after intravenous or oral administration of MT at a dose of 2 mg/kg (Yang et al., 2010). However, in recent years, several studies have demonstrated that MT can lead to DILI, reproductive toxicity, and neurotoxicity (Wang et al., 2017). MT has been demonstrated to induce hepatotoxicity through inhibiting the Nrf2 pathway and stimulating the reactive oxygen species (ROS)-mediated mitochondrial apoptosis pathway (You et al., 2019). Nevertheless, the mechanisms of MT in liver protection and hepatotoxicity are continually being improved and elucidated (Figure 1).

Calcium ion (Ca^2+^), a multifunctional intracellular messenger, affects cellular metabolism, energy generation, and intracellular homeostasis under physiological conditions. Extracellular stress stimulation on the cell membrane could enhance Ca^2+^ influx, and increased cytoplasmic Ca^2+^ would be transported into the Endoplasmic reticulum (ER) lumen and stored via cross specific Ca^2+^ ion channels, such as sarcoendoplasmic reticulum calcium transport ATPase (SERCA) (Periasamy and Kalyanasundaram, 2007; Chemaly et al., 2018). Loss of Ca^2+^ homeostasis and irregular Ca^2+^ channels on the cell membrane, ER, and mitochondria might cause ER stress and modify the mitochondrial membrane potential, raising total ROS in hepatocytes (Kaufman and Malhotra, 2014; Zeeshan et al., 2016). Based on the current literature, distinct MT concentration gradients can influence diverse SERCA responses on the ER, regulate mitochondrial activity, and balance intracellular Ca^2+^ levels to alleviate or promote hepatocyte stress (Gao et al., 2019).

Previous studies have demonstrated that MT exhibits both hepatoprotective effects and the potential to cause liver damage, yet the underlying mechanisms remain unclear. Therefore, the objective of this study is to conduct a systematic review and meta-analysis to investigate the impact of MT on LI and elucidate the dynamic processes through which MT leads to liver protection and hepatotoxicity. Additionally, this study aims to explore the role of Ca^2+^ in these processes, offering innovative insights into the mechanisms involved.

## 2 Methods

### 2.1 Registration of the meta-analysis

The meta-analysis followed the PRISMA 2020 guidelines and has been submitted to the International Platform of Registered Systematic Review and Meta-analysis Protocols (INPLASY) database (https://inplasy.com/). The registration number for this submission is INPLASY202340114.

### 2.2 Data sources and search strategy

The retrieved databases included four English databases and four Chinese databases according to the five articles (Ju et al., 2018; Xiong et al., 2019; Liu et al., 2021; Luo et al., 2021; Zheng et al., 2021). The four English databases were: PubMed, Web of science, Cochrane library, Embase. And the four Chinese databases: China National Knowledge Infrastructure, WanFang Med Online, China Science and Technology Journal Database, and China Biomedical Literature Service System. The literature search in this study encompassed all pertinent literature up until May 2023.

**FIGURE 2:**
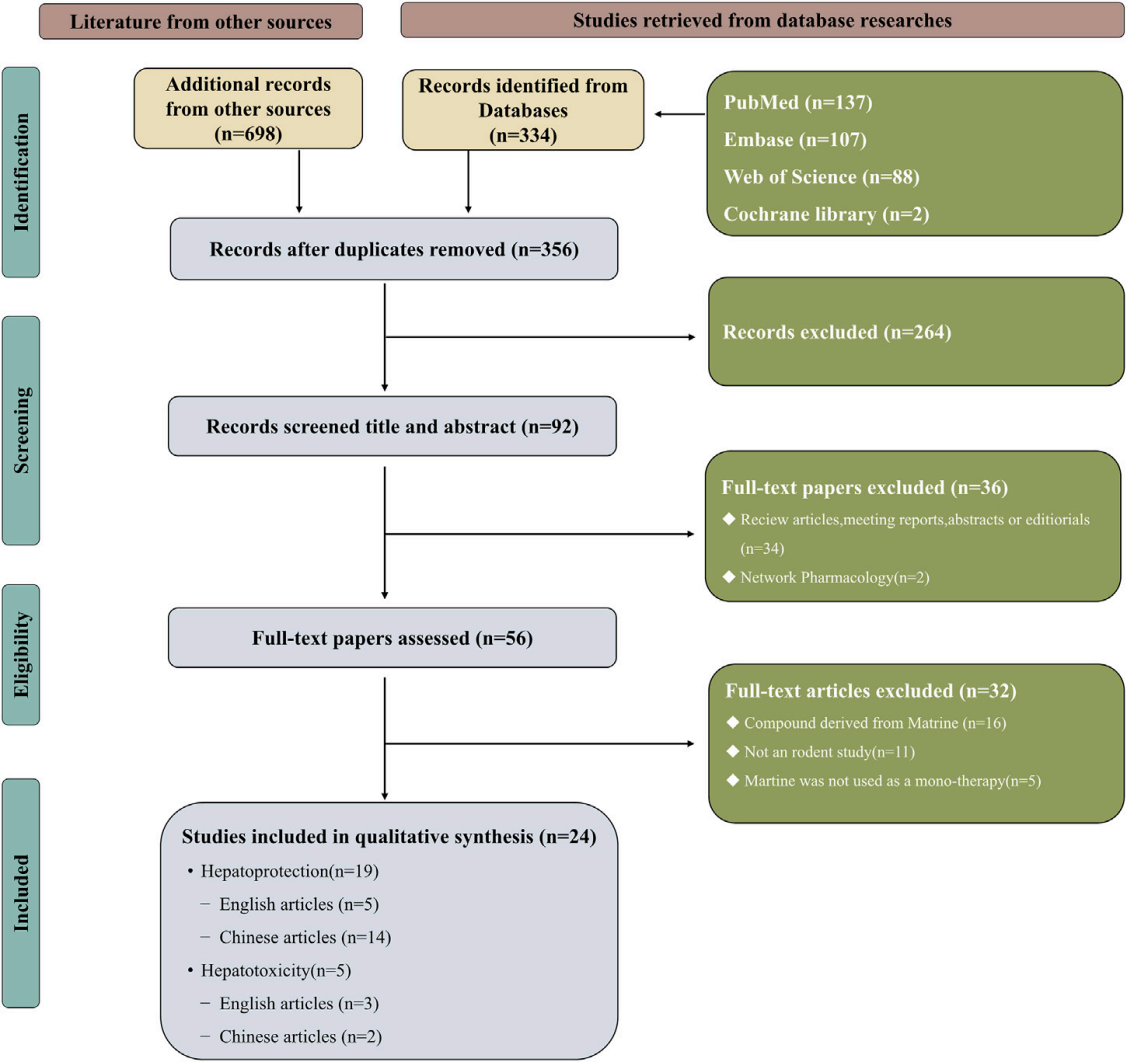
Selection of studies for the meta-analysis.

The search terms were “Matrine,” “liver injury,” “hepatoprotection,” and “hepatotoxicity”. (Figure 2 and Supplementary Table S1).

### 2.3 Included criteria and excluded criteria

Considering the difference between hepatotoxicity and hepatoprotection of MT, this article has formulated appropriate included and excluded criteria to address these dual effects.

#### 2.3.1 Included criteria

Research of hepatoprotection needs to meet the following requirements: 1) Subjects: the study involved rats or mice as the population. 2) Control(C) group and intervention(I) group: each study included at least 1 LI group as the C group and 1 MT group as the I group. 3) The I groups were comprised of LI models and received MT monotherapy exclusively. The C groups consisted of LI models that either received no treatment or received non-functional intervention. 4) The indicators of the studies should encompass AST, ALT, MDA, SOD, serum TG, serum TC, IL-6, CAT, and TNF-α, either in their entirety or partially. 5) The quality evaluation results were above 5 points.

Research of hepatotoxicity needs to meet the following requirements: 1) Subjects: the study involved rats or mice as the population. 2) C group and I group: each study included at least 1 LI group as the C group and 1 MT group as the I group. 3). The I groups were comprised of normal animals and received MT monotherapy exclusively. The C groups consisted of normal animals that either received no treatment or received non-functional intervention. 4) The indicators of the studies should encompass AST and ALT, either in their entirety or partially. 5) The quality evaluation results were above 5 points.

#### 2.3.2 Excluded criteria

Excluded criteria for the research of hepatoprotection: 1) LI rats or mice were not selected as animal subjects for hepatoprotection studies. 2) The experiments did not form controls. 3) The I group did not receive MT monotherapy. The C group used functional drugs (including Western medicines, TCMs and integrative medicines) as interventions, and could not provide specific outcome indices. 4) The common indicators of LI were not included in the study. 5) Quality evaluation results below 5 points.

Excluded criteria for the research of hepatotoxicity: 1) Normal rats or mice were not selected as subjects for hepatotoxicity studies. 2) The experiments did not form controls. 3) The I group did not receive MT monotherapy. The C group used functional drugs (including Western medicines, TCMs and integrative medicines) as interventions, and could not provide specific outcome indices. 4) The common indicators of LI were not included in the study. 5) Quality evaluation results below 5 points.

### 2.4 Data extraction

Two researchers extracted the following data from the included articles: 1) The first author’s name and publication year; 2) Basic animal characteristics: including the number, species (mice or rats), strain, sex, and weight; 3) Modeling details: including the method of modeling and criteria for successful modeling; 4) Specific intervention methods: including the drug used, dosage, and frequency of administration; 5) Outcome measures. (Table 1).

**TABLE 1:** The key characteristics of all 24 Studies.

Author(s)/Year	Species	Gender (M/F)	Weight of the animal	Sample size (n)MT/Model	Drug dosage	Treatment courses	Main outcome indicators
Song2009	Kunming mice	NM	18–22 g	12/12	MT: MT,62.5 mg/kg	10 days	ALT, AST
					Mod: No drugs		
Liang2015	ICR mice	Female and male	18–22 g	10/10	MT: MT,40 mg/kg	60 days	ALT, AST
					Mod: Equal volume water		
Gu2019	C57BL/6 mice	Male	18–20 g	11/10	MT: MT,69.1 mg/kg	90days	ALT, AST
					Mod: Distilled water (constant volume)		
Liu2020	BALB/c mice	Male	17.7–18.1 g	10/10	MT: MT,100 mg/kg	1 week	ALT, AST
					Mod: Normal saline		
Rao2022	ICR mice	Male	18–22 g	10/10	MT: MT,60 mg/kg	2 weeks	ALT, AST
					Mod: Normal saline		
Li2005	NIH mice	Male	18–22 g	10/10	MT: Con A,20 mg/kg + MT,25 mg/kg	3 days	ALT, TNF-α
					Mod: Con A,20 mg/kg		
Liu2008	Sprague Dawley rats	Male	210–230 g	92/92	MT: HIRI + MT,60 mg/kg	1 day	ALT, AST, IL-6, TNF-α
					Mod: HIRI		
Zhou2009	NIH mice	Male	18–22 g	13/11	MT: Con A,20 mg/kg + MT,25 mg/kg	5 days	ALT, AST
					Mod: Con A,20 mg/kg		
Yang2013	Sprague Dawley rats	Male	180–220 g	24/24	MT: 2-AAF, 15 mg/kg + MT,20 mg/kg	3 weeks	ALT
					Mod: 2-AAF, 15 mg/kg		
Shi2013	C57BL/6 mice	Female	NM	8/8	MT: CCl_4_,0.6 mL/kg + MT,30 mg/kg	6 weeks	ALT
					Mod: CCl_4_,0.6 mL/kg		
Zhang2013	Wistar rats	Male	180–200 g	6/6	MT: High-fructose diet + MT,160 mg/kg	4 weeks	ALT, AST, Serum TG, TNF-α, CAT, MDA, SOD
					Mod: High-fructose diet		
Gao2013	Sprague Dawley rats	Female and male	100–140 g	12/12	MT: Chinese liquor,43% vol + MT, 50 mg/kg	30 days	ALT, AST, Serum TG, Serum TC, CAT, MDA, SOD
					Mod: Chinese liquor,43% vol		
Tang2013	Wistar rats	Female and male	113–118 g	10/10	MT: High fatty diet + MT,36 mg/kg	30 days	Serum TG, Serum TC
					Mod: High fatty diet		
Wu2014	Kunming mice	Male	18–22 g	10/10	MT: ethanol,5 g/kg + MT,80 mg/kg	6 days	ALT, AST, MDA, SOD
					Mod: ethanol,5 g/kg		
Zhu2015	Sprague Dawley rats	Male	200–250 g	15/15	MT: HIRI + MT,30 mg/kg	4 h	ALT, AST, TNF-α
					Mod: HIRI		
Zhao2015	Wistar rats	Male	180–220 g	12/12	MT: CCl_4_,1 mL/kg + MT,10 mg/kg	1 week	ALT, AST, TNF-α
					Mod: CCl_4_,1 mL/kg		
Li2016	Sprague Dawley rats	Female and male	100–140 g	12/12	MT: Chinese liquor,43% vol + MT, 100 mg/kg	30 days	ALT, AST, Serum TG, Serum TC, CAT, MDA, SOD
					Mod: Chinese liquor,43% vol		
Guo2017	Sprague Dawley rats	Male	180–220 g	8/8	MT: CCl_4_,3 mL/kg + MT,72.8 mg/kg	3 weeks	ALT, AST
					Mod: CCl_4_,3 mL/kg		
Gao2018	C57BL/6 mice	Male	NM	10/10	MT: High-fat diet + MT,10 mg/kg	7 weeks	ALT, AST, Serum TG, Serum TC, TNF-α
					Mod: High-fat diet		
Bai2018	Sprague Dawley rats	Male	200–250 g	10/10	MT: HIRI + MT,50 mg/kg	4 h	ALT, AST, TNF-α
					Mod: HIRI		
Khan2019	BALB/c mice	Male	24–35 g	5/5	MT: CCL_4_,1 mL/kg + MT,50 mg/kg	1 day	ALT, AST, MDA
					Mod: CCL_4_,1 mL/kg		
Yuan2020	Sprague Dawley rats	Male	200–250 g	6/6	MT: HIRI + MT,40 mg/kg	1 week	ALT, AST, MDA, SOD
					Mod: HIRI		
Chang2021	C57BL/6 mice	Male	18–22 g	8/8	MT: acetaminophen,400 mg/kg + MT,2.8 mg/kg	1 week	ALT, AST, TNF-α, MDA, SOD
					Mod: acetaminophen, 400 mg/kg		
Du2021	Kunming mice	Male	18–22 g	6/6	MT: ethanol,5.4 g/kg + MT,2.8 mg/kg	2 weeks	ALT, AST, CAT, MDA, SOD
					Mod: ethanol,5.4 g/kg		

**Abbreviations:** Green area: in the matter of hepatotoxicity of MT (n = 5); Yellow area: in the matter of hepatoprotection of MT(n = 19) **NM**, not mentioned; **ICR**, Institute of Cancer Research; **MT**, matrine; **Mod**, model; **2‐AAF**, N‐2‐acetylaminofluorene; **HIRI**, hepatic ischemia-reperfusion injury (clip-closed portal vein and hepatic artery followed by reperfusion); **ALT**, alanine aminotransferase; **AST**, aspartate aminotransferase; **IL‐6**, interleukin‐6; **TNF-α**, tumor necrosis factor alpha; Serum TG, serum triglyceride; **Serum TC**, serum cholesterol; **CAT**, catalase; **SOD**, superoxide dismutase; **MDA**, malondialdehyde.

Regarding the preset indicators, we recorded only the highest dose group in the gradient dosages. For the experiments that observed data from multiple time points, only the last were recorded. We collected the experimental data by Universal Desktop Ruler and calculated the mean and standard deviation (SD) of the continuous variables. Results of the measurements were displayed in graphics rather than digital text.

### 2.5 Risk of bias and quality of evidence

**FIGURE 3:**
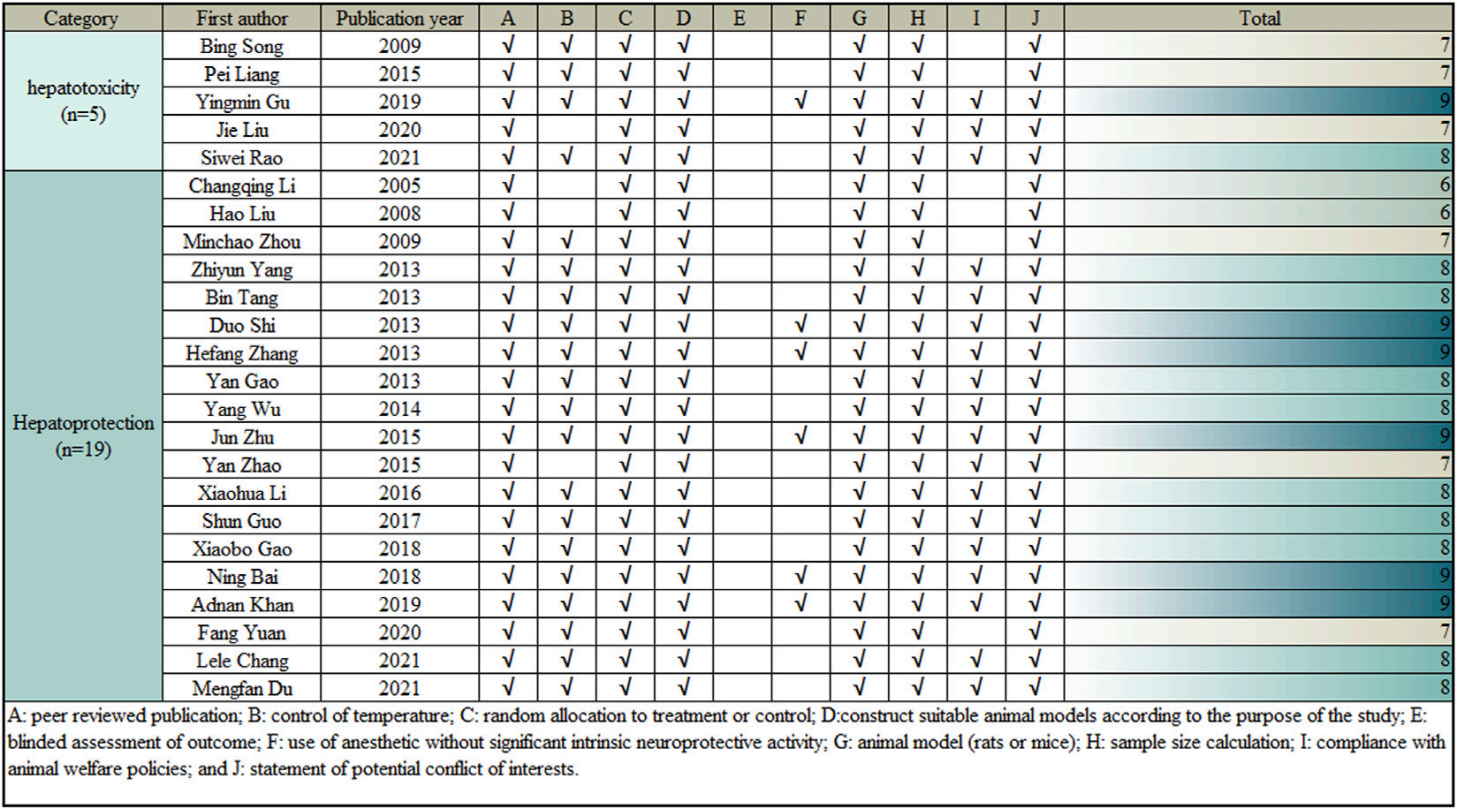
Risk of bias and quality assessment scores in each study.

The CAMARADES (Collaborative Approach to Meta-Analysis and Review of Animal Data from Experimental Studies) 10-point scoring scale, an internationally recognized criteria published in 2004, is utilized to assess and calculate a methodological quality score (Macleod et al., 2004). Two researchers made an independently assessment of the methodological quality of the surveys. The quality measures were changed in accordance with the study’s specificity. When there was a disagreement in the evaluation, the correspondence author came to an agreement or used arbitration. The specific methods were also provided in Figure 3.

### 2.6 The dose-time-effect relationship and mechanism analysis of MT

To visualize the dose-time-effect relationship for hepatoprotection and hepatotoxicity of MT, this study unified the time units of all experiments into weeks (W), as well as constructed 3D maps and radar charts. In addition, the regulatory mechanism of MT role in the literature is summarized.

### 2.7 Quantitative synthesis and statistical analyses

Statistical analysis of indicators in this study was conducted using STATA 16.0 software. When the results are statistically significant, the *p*-value should be less than 0.05 (*p* < 0.05). Results were quantified using the standardized mean differences (SMD) and accompanying 95% confidence intervals (95% CI). The I-squared (I^2^) test was used to assess the degree of heterogeneity and consistency between research (random-effects model [I^2^>50%] or fixed-effects model [I^2^ ≤ 50%]). Results were deemed to exhibit significant heterogeneity when I^2^ exceeded 50%. Investigators conducted subgroup analyses for animal species (rat, mouse), dose administered (low (L)≤25 mg/kg, 25<medium (M)≤50 mg/kg, high (H)>50 mg/kg), and time of administration (<4w, ≥4w) in order to identify the source of heterogeneity. To establish whether the findings were trustworthy enough to draw inferences, sensitivity analysis was done.

### 2.8 Molecular docking

The compounds and ligands were obtained from the PubChem database (https://pubchem.ncbi.nlm.nih.gov) and RCSB Protein Data Bank database (https://www.rcsb.org/structure). Molecular docking was performed using AutoDockTools 1.5.6 and AutoDock Vina 4.2. Here is a brief summary of the docking process.

1) The structure of MT was downloaded from the PubChem database. It was then converted into a 3D structure using ChemDraw software to minimize the structural energy. The 3D structure was calculated using AutoDockTools 1.5.6 software and saved as a pdbqt file. 2) The ligands were obtained from the RCSB protein bank. They were imported into PyMOL, dehydrated, hydrogenated, and prepared for ligand separation. The docking grid box was constructed in AutoDockTools 1.5.6 at the active site for each target protein and saved in pdbqt format. 3) AutoDock Vina 1.1.2 was used for molecular docking of the potential targets and active compounds, as well as to evaluate free binding energies. 4) PyMOL 2.6 and Discovery Studio 2019 were utilized for visualizing and analyzing interactions.

## 3 Results

### 3.1 Comprehensive literature review and selection

By using keywords, 1,032 articles in all could be found (334 articles from the four English databases and 698 articles from the four Chinese databases). After eliminating 676 duplicate articles, the researchers further examined the rest 356 articles. Depending upon the inclusion and exclusion criteria, the researchers excluded 264 articles after reviewing the titles and abstracts. And 34 articles on MT reviews, conference reports, abstracts or editorials and web pharmacology were subsequently eliminated from consideration. The remaining 32 articles were excluded after reviewing the full text. This meta-analysis eventually comprised 24 publications, 16 of which were in Chinese (Li et al., 2005; Liu et al., 2008; Song et al., 2009; Zhou et al., 2009; Gao et al., 2013; Tang et al., 2013; Wu et al., 2014; Liang et al., 2015; Zhao, 2015; Zhu et al., 2015; Li et al., 2016; Guo et al., 2017; Bai et al., 2018; Yuan and Yang, 2020; Chang et al., 2021; Du et al., 2021) and others were in English (Shi et al., 2013; Yang et al., 2013; Zhang et al., 2013; Gao et al., 2018; Gu et al., 2019; Khan et al., 2019; Liu et al., 2020; Rao et al., 2022). Figure 2 illustrates a flowchart of the study selection process.

### 3.2 Study quality

A modified 10-item CAMARADES checklist was used to assess the methodological quality of the included publications. Peer-reviewed articles were among the criteria; temperature management; construction of appropriate rodent models according to the study objectives; experimental animals were randomly assigned to treatment or control groups; blinded assessment of outcomes; explicit presentation of the use of anaesthetics without significant intrinsic neuroprotective activity; sample size calculations; compliance with animal welfare policies; and avoidance of potential conflicts of interest. All 24 articles used appropriate rodent models and reasonable groupings, all clearly reported sample sizes for each group and competing interests, and all were published in peer-reviewed publications. However, only 6 articles explicitly reported the use of anaesthetics with no apparent intrinsic neuroprotective activity, 4 did not mention temperature control in the experiments, 5 did not mention animal welfare policies, and no studies assessed outcomes blinded. The included articles’ overall quality ratings ranged from 6 to 9. Two of the 24 articles received a score of 6 (8.33%), six received a score of 7 (25.00%), ten received a score of 8 (41.67%), and six received a score of 9 (25.00%). The methodological quality of each selected article is demonstrated in Figure 3.

### 3.3 Basic information and features of the articles included

The 24 papers had enough information to conduct a meta-analysis. These trials involved a total of 657 rodents, 330 of which were divided into the treatment group and the others were control group (Table 1).

Based on their biological traits, the animals used in the included researches were roughly categorized. The creatures were categorized into seven groups based on their species: 8.52% (56/657) Kunming mice, 6.09% (40/657) ICR mice, 11.11% (73/657) C57BL/6 mice, 4.57% (30/657) BALB/c mice, 54.49% (358/657) Sprague Dawley Rats, 8.52% (56/657) Wistar rats and 6.70% (44/657) NIH mice. 63.01% (414/657) of rodents were rats, and 36.99% (243/657) of rodents were mice. The percentage of female and male rodents was 9.13% (60/657) and 87.21% (573/657) respectively regarding sex categorization, while 3.65% (24/657) of the rodents’ sexes were unknown. Furthermore, according to the quality assessment scores, 25.00% (6/24) had 9 points, 41.67 (10/24) had 8 points, 25.00% (6/24) had 7 points, and 8.33% (2/24) had 6 points. Regarding the intervention time of MT, all experiments were divided into two subgroups: 76.10% (500/657) <4W groups and 23.90% (157/657) ≥4W groups. And the dosage of each experiment was divided into three groups: 24.96% (164/657) L-dosage groups, 23.14% (152/657) M-dosage group and 51.90% (341/657) H-dosage group (Supplementary Figure S1).

Across the studies, the weight of the animals included in the analysis varied from 17.7 g to 250 g, with a total number of examinations ranging from 10 to 184. The daily dosage of MT administered ranged from 2.8 mg/kg to 160 mg/kg, and the frequency of administration varied from a single dose to a maximum of 90 days.

### 3.4 Effects of MT on LI

The levels of ALT, AST, TNF-α and SOD which were the primary outcomes were assessed after MT therapy as well as the levels of MDA, IL-6, serum TG, serum TC and CAT were also changed by MT (Supplementary Tables S2, S3). Liver tissues from animals with LI exhibited significant inflammatory cell infiltration, hepatocyte swelling, vacuolar degeneration, and hepatocellular necrosis, as evidenced by H&E staining. The pathogenic alterations were significantly improved with MT treatment at dosage of 1.4–100 mg/kg, but the most effective dosage was the medium (25–50 mg/kg/d).

#### 3.4.1 MT can improve the primary outcomes of LI

##### 3.4.1.1 ALT levels

**FIGURE 4:**
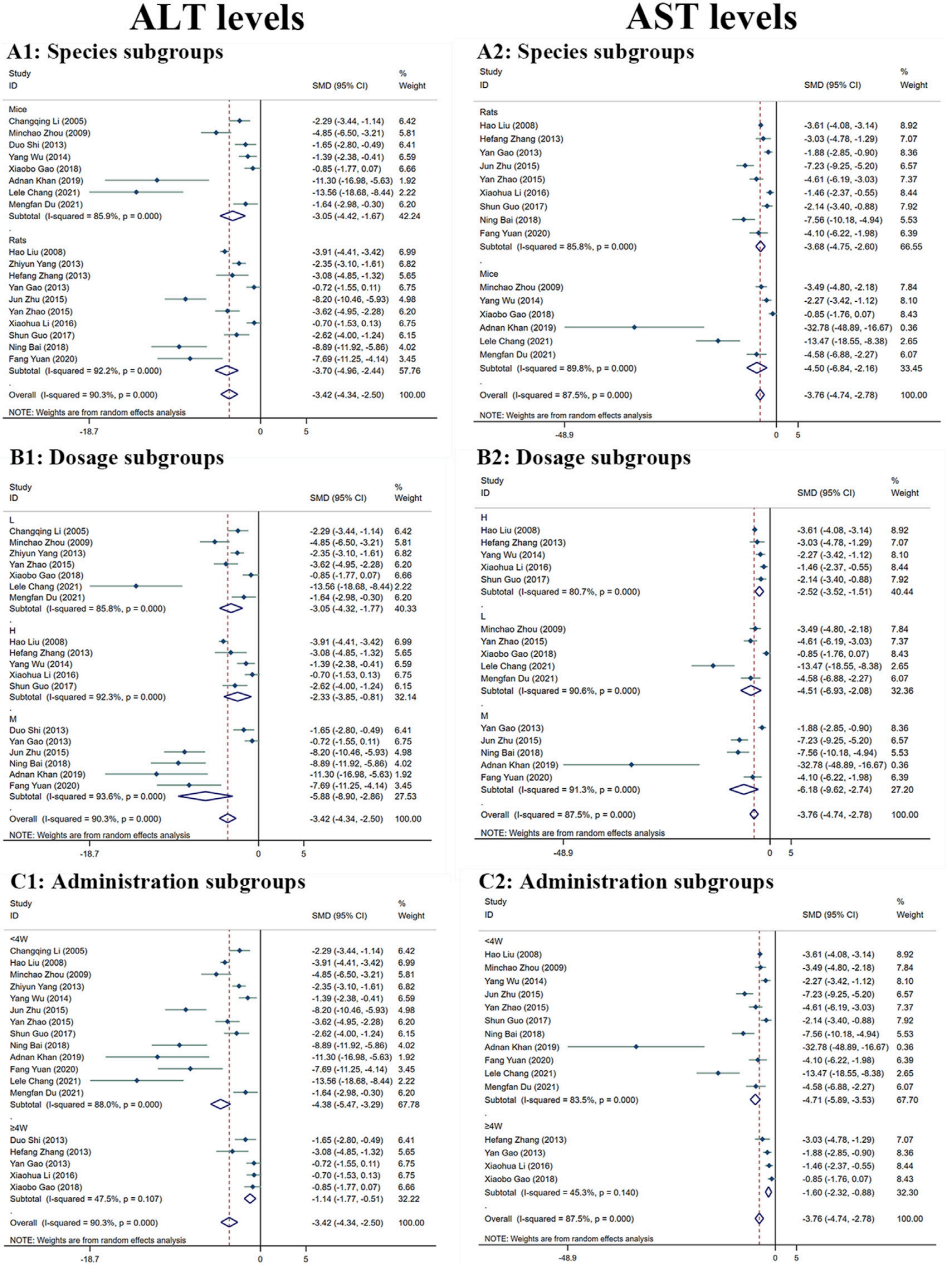
Forest plot (effect size and 95% CI) summarising the effects of MT on ALT **(A1–C1)** and AST **(A2–C2)** levels in hepatoprotection. **(A)** Rat and mice subgroups; **(B)** L, M and H dosage subgroups; **(C)** < 4 weeks and ≥4 weeks of administration subgroups.

Because there was considerable heterogeneity (I^2^ > 50%), we performed a random-effects analysis. The findings revealed that the ALT levels were significantly reduced in the MT groups compared to the LI model groups (n = 532; *95% CI* [−4.34, −2.50]; *SMD* = |−3.42| > 1; *I* ^*2*^ = 90.30%; *p* < 0.0001) (Figure 4).

##### 3.4.1.2 AST levels

The random-effect analysis was used for further analysis as the significant heterogeneity (I^2^ > 50%). The random-effect analysis revealed that the AST levels between the MT and LI model groups were significantly different. The levels of AST were shown to be reduced by MT (n = 448; *95% CI* [-4.74, −2.78]; *SMD* = |−3.76| > 1; *I* ^*2*^ = 87.50%; *p* < 0.0001) (Figure 4).

##### 3.4.1.3 SOD levels

**FIGURE 5:**
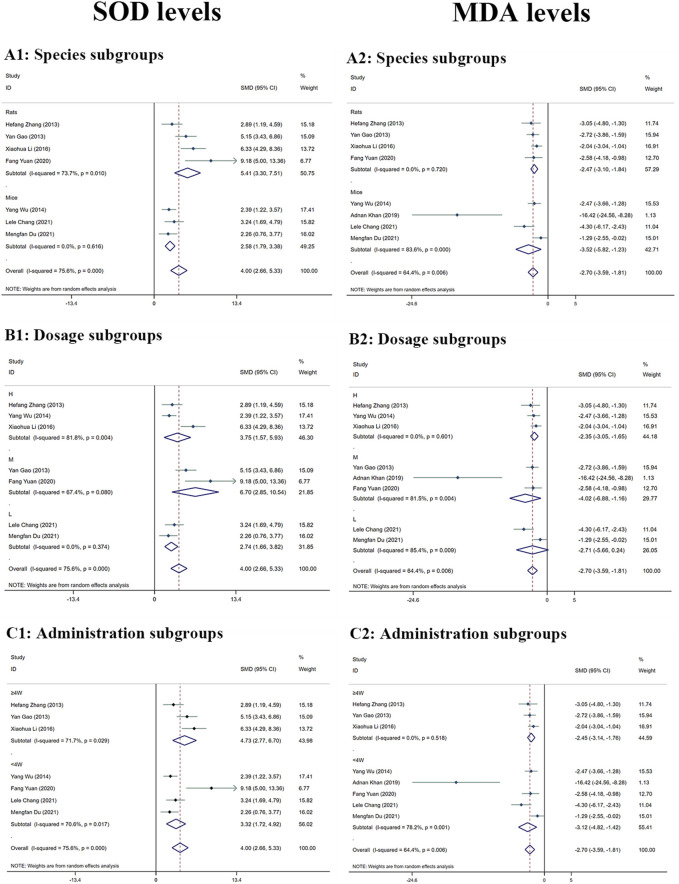
Forest plot (effect size and 95% CI) summarising the effects of MT on SOD **(A1–C1)** and MDA **(A2–C2)** levels in hepatoprotection. **(A)** Rat and mice subgroups; **(B)** L, M and H dosage subgroups; **(C)** < 4 weeks and ≥4 weeks of administration subgroups.

Significant heterogeneity (I^2^ > 50%) was observed, and a random-effects analysis was conducted. The results indicated that the amounts of SOD protein in the MT groups were substantially greater than in the LI model groups (n = 120; *95% CI* [2.66,5.33]; *SMD* = |4.00| > 1; *I* ^*2*^ = 75.60%; *p* < 0.0001) (Figure 5).

##### 3.4.1.4 MDA levels

The MDA levels in the mammalian models in the included study varied according to random-effects analyses. MDA levels in the MT groups were considerably lower than in the model groups (n = 130; *95% CI* [-3.59, −1.81]; *SMD* = |-2.70| > 1; *I* ^*2*^ = 64.40%; *p* < 0.0001) (Figure 5).

#### 3.4.2 MT can administer the secondary outcomes of LI

##### 3.4.2.1 Serum TG levels

The random-effects analysis showed that there were differences in serum TG levels between the MT and LI model groups. The MT groups had significantly lower levels of serum TG compared to the LI model group (n = 100; *95% CI* [-2.70, −0.67]; *SMD* = |-1.68| > 1; *I* ^*2*^ = 77.00%; *p* = 0.001) (Supplementary Figure S2).

##### 3.4.2.2 Serum TC levels

In accordance with the random-effects analysis, the animal models in the included research had different serum TC levels. The MT groups had considerably lower serum TC levels than the model groups (n = 88; *95% CI* [-2.59, −0.78]; *SMD* = |-1.69| > 1; *I* ^*2*^ = 68.60%; *p* < 0.0001) (Supplementary Figure S2).

##### 3.4.2.3 IL-6 levels

A random-effects analysis found notable IL-6 levels discrepancies between the MT and LI model groups. When compared to the LI model groups, IL-6 levels were substantially lower in the MT groups (n = 254; *95% CI* [-4.87, −2.67]; *SMD* = |-3.77| > 1; *I* ^*2*^ = 66.80%; *p* < 0.0001) (Supplementary Figure S3).

##### 3.4.2.4 TNF-α levels

Because there was considerable heterogeneity (I^2^ > 50%), we performed a random-effects analysis for additional research. Regarding the random-effect analysis, the MT and LI model groups showed significantly different levels of TNF-α. TNF-α levels was observed to be reduced by MT (n = 326; *95% CI* [-5.50, −1.95]; *SMD* = |-3.72| > 1; *I* ^*2*^ = 94.60%; *p* < 0.0001) (Supplementary Figure S3).

##### 3.4.2.5 CAT levels

The animal models in the included studies showed several differences between the MT and LI model groups in CAT levels, according to random-effect analysis. The MT groups had significantly higher levels of CAT than model groups (n = 72; *95% CI* [2.23,3.62]; *SMD* = |2.93| > 1; *I* ^*2*^ = 0.00%; *p* < 0.0001) (Supplementary Figure S3).

#### 3.4.3 Subgroup analysis

##### 3.4.3.1 Subgroup analysis of ALT levels

In comparison to the LI model groups, the levels of ALT were found to be significantly reduced in the MT groups. MT was effective in both rats (n = 394; 95% *CI* [-4.96, −2.44]; *SMD* = |–3.70| > 1; *I* ^*2*^ = 92.20%; *p* < 0.0001) and mice (n = 138; 95% *CI* [-4.42, −1.67]; *SMD* = |–3.05| > 1; *I* ^*2*^ = 85.90%; *p* < 0.0001) (Figure 4A1). The ALT levels were found to reduced most by MT in the M-dosage subgroups (n = 112; 95% *CI* [-8.90,-2.86]; *SMD* = |–5.88| > 1; *I* ^*2*^ = 93.60%; *p* < 0.0001) than H-dosage (n = 256; 95% *CI* [-3.85,-0.81]; *SMD* = |–2.33| > 1; *I* ^*2*^ = 92.30%; *p* = 0.003) and L-dosage subgroups (n = 164; 95% *CI* [-4.32,-1.77]; *SMD* = |–3.05| > 1; *I* ^*2*^ = 85.80%; *p* < 0.0001) (Figure 4B1). Furthermore, it worked in both ‘≥4W’ subgroups (n = 96; 95% *CI* [-1.77, −0.51]; *SMD* = |–1.14| > 1; *I* ^*2*^ = 47.50%; *p* < 0.0001) and ‘<4W’ subgroups (n = 436; 95% *CI* [-5.47, −3.29]; *SMD* = |–4.38| > 1; *I* ^*2*^ = 88.00%; *p* < 0.0001), but the lower levels were in the ‘<4W’ subgroups (Figure 4C1).

##### 3.4.3.2 Subgroup analysis of AST levels

Compared with the LI model group, the levels of AST in MT groups were significantly lower. MT reduced substantially the AST levels in both rats’ subgroups (n = 346; 95% *CI* [-4.75, −2.60]; *SMD* = |–3.68| > 1; *I* ^*2*^ = 85.80%; *p* < 0.0001) and mice subgroups (n = 102; 95% *CI* [-6.84, −2.16]; *SMD* = |–4.50| > 1; *I* ^*2*^ = 89.80%; *p* < 0.0001) (Figure 4A2). MT had the most significant effect in the M-dosage subgroups (n = 96; 95% *CI* [-9.62,-2.74]; *SMD* = |–6.18| > 1; *I* ^*2*^ = 91.30%; *p* < 0.0001) than the other two subgroups (H-dosage subgroups: n = 256; 95% *CI* [-3.52,-1.51]; *SMD* = |–2.52| > 1; *I* ^*2*^ = 80.70%; *p* < 0.0001) (L-dosage subgroups: n = 96; 95% *CI* [-6.93,-2.08]; *SMD* = |-4.51| > 1; *I* ^*2*^ = 90.60%; *p* < 0.0001) (Figure 4B2). The levels of AST were decreased in the both two time-subgroups, but the lower groups were ‘<4W’ subgroups (‘≥4W’ subgroups: n = 80; 95% *CI* [-2.32,-0.88]; *SMD* = |-1.60| > 1; *I* ^*2*^ = 45.30%; *p* < 0.0001) (‘<4W’ subgroups: n = 368; 95% *CI* [-5.89,-3.53]; *SMD* = |-4.71| > 1; *I* ^*2*^ = 83.50%; *p* < 0.0001) (Figure 4C2).

##### 3.4.3.3 SOD levels subgroup analysis

The amount of SOD was significantly greater in the groups treated with MT than in the LI model groups. The levels of SOD were increased by MT in both rats subgroups (n = 72; 95% *CI* [3.30,7.51]; *SMD* = |5.41| > 1; *I* ^*2*^ = 73.70%; *p* < 0.0001) and mice subgroups (n = 48; 95% *CI* [1.79,3.38]; *SMD* = |2.58| > 1; *I* ^*2*^ = 0.00%; *p* < 0.0001) (Figure 5A1). The levels of SOD of M-dosage subgroups (n = 36; 95% *CI* [2.85,10.54]; *SMD* = |6.70| > 1; *I* ^*2*^ = 67.40%; *p* = 0.001) were the highest by MT than H-dosage subgroups (n = 56; 95% *CI* [1.57,5.93]; *SMD* = |3.75| > 1; *I* ^*2*^ = 81.80%; *p* = 0.001) and L-dosage subgroups (n = 28; 95% *CI* [1.66,3.82]; *SMD* = |2.74| > 1; *I* ^*2*^ = 0.00%; *p* < 0.0001) (Figure 5B1). Furthermore, it worked in both ‘≥4W’ subgroups (n = 60; 95% *CI* [2.77,6.70]; *SMD* = |4.73| > 1; *I* ^*2*^ = 71.70%; *p* < 0.0001) and ‘<4W’ subgroups (n = 60; 95% *CI* [1.72,4.92]; *SMD* = |3.32| > 1; *I* ^*2*^ = 70.60%; *p* < 0.0001), but the higher levels were in the ‘<4W’ subgroups (Figure 5C1).

##### 3.4.3.4 Subgroup analysis of MDA levels

The MDA levels in the MT groups were lower than those in the LI model groups. The MDA levels were decreased by MT in both rats subgroups (n = 72; 95% *CI* [-3.10, −1.84]; *SMD* = |-2.47| > 1; *I* ^*2*^ = 0.00%; *p* < 0.0001) and mice subgroups (n = 58; 95% *CI* [-5.82, −1.23]; *SMD* = |-3.52| > 1; *I* ^*2*^ = 83.60%; *p* = 0.003) (Figure 5A2). MT reduced the MDA levels most in the M-dosage subgroups (n = 46; 95% *CI* [-2.55,-0.02]; *SMD* = |-4.02| > 1; *I* ^*2*^ = 81.50%; *p* = 0.006) among the time subgroups (H-dosage subgroups: n = 56; 95% *CI* [-3.05,-1.65]; *SMD* = |–2.35| > 1; *I* ^*2*^ = 0.00%; *p* < 0.0001) (L-dosage subgroups: n = 28; 95% *CI* [-5.66,0.24]; *SMD* = |-4.51| > 1; *I* ^*2*^ = 85.40%; *p* = 0.071) (Figure 5B2). Moreover, it substantially lowered MDA levels in both ‘≥4W’ subgroups (n = 60; 95% *CI* [-3.14, −1.76]; *SMD* = |-2.45| > 1; *I* ^*2*^ = 0.00%; *p* < 0.0001) and ‘<4W’ subgroups (n = 70; 95% *CI* [-4.82, −1.42]; *SMD* = |-3.12| > 1; *I* ^*2*^ = 78.20%; *p* < 0.0001) (Figure 5C2).

#### 3.4.4 Sensitivity analysis and publication bias of outcome indicators

The sensitivity of ALT, AST, SOD, and MDA levels in detecting LI in mouse models did not differ significantly. To identify publication bias, we used the |t|-value and conducted Egger’s test. The |t|-values of these four factors did not indicate any publication bias in LI research (Supplementary Figures S4, S5).

### 3.5 The toxic effects of MT on liver

ALT and AST levels were examined as significant main indicators of toxic effects of MT on liver in five investigations. All the five investigations showed that MT can significantly increase ALT and AST levels. According to the results, MT may increase hepatotoxicity by influencing ALT and AST levels (Supplementary Table S4). H&E staining of normal animal liver tissues revealed significant hepatotoxicity with inflammatory cell infiltration, cell edema, cytoplasmic loosening and vacuolar degeneration of cytoplasm. Significant pathogenic alterations occurred with the intervention of MT at 10–69.1 mg/kg, but the most toxic dosage was the 30–62.5 mg/kg/d.

#### 3.5.1 MT can affect the main indicators of liver function

##### 3.5.1.1 ALT levels

**FIGURE 6:**
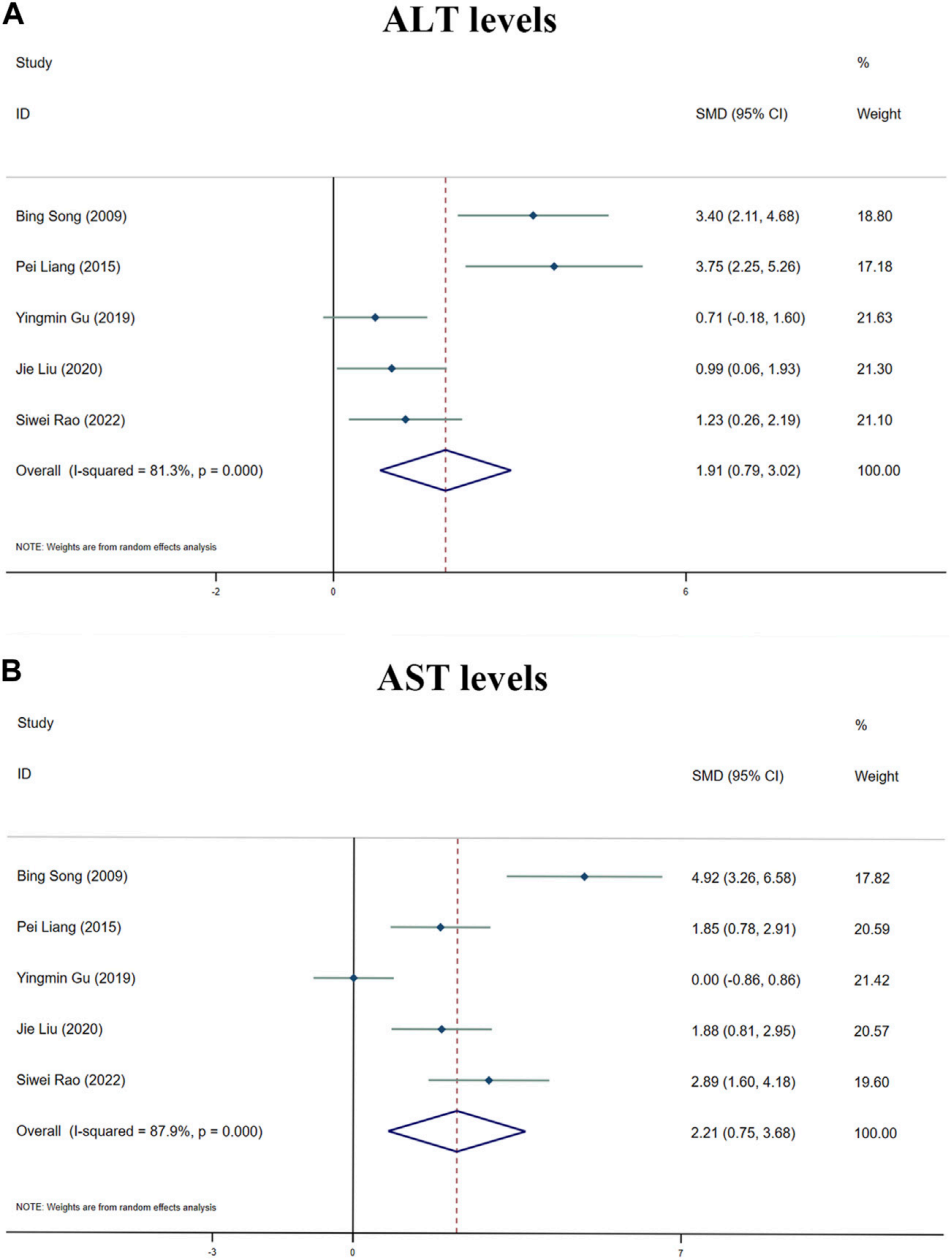
Forest plot (effect size and 95% CI) summarising the effects of MT on hepatotoxicity. **(A)** ALT levels; **(B)** AST levels.

Because of the considerable heterogeneity (I^2^ > 50%), the random-effects analysis was utilized for further investigation. The random-effects analysis revealed that the ALT levels between the MT and control groups were significantly different. The level of ALT was shown to be elevated by MT (n = 105; *95% CI* [0.79, 3.02]; *SMD* = |1.91| > 1; *I* ^*2*^ = 81.30%; *p* < 0.0001) (Figure 6A).

##### 3.5.1.2 AST levels

The random-effect analysis revealed that the AST levels between the MT and control groups were significantly different. The level of AST was shown to be elevated by MT (n = 105; 95*% CI* [0.75, 3.68]; *SMD* = |2.21| > 1; *I* ^*2*^ = 87.90%; *p* < 0.0001) (Figure 6B).

### 3.6 Dose–time–effect/dose–time–toxicity relationship

**FIGURE 7:**
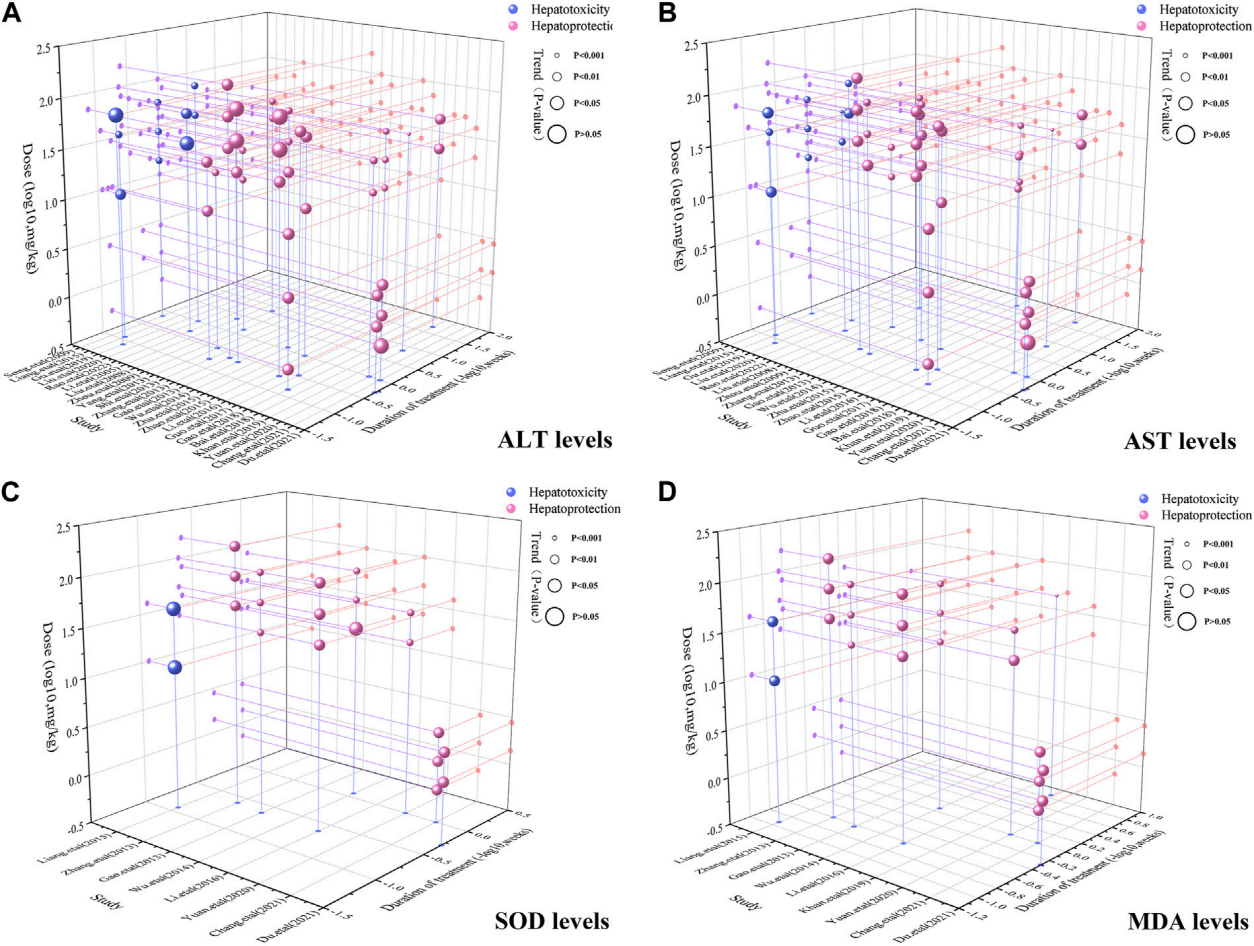
3D maps of dose–time–effect relationship. **(A)** ALT levels; **(B)** AST levels; **(C)** SOD levels; **(D)** MDA levels.

**FIGURE 8:**
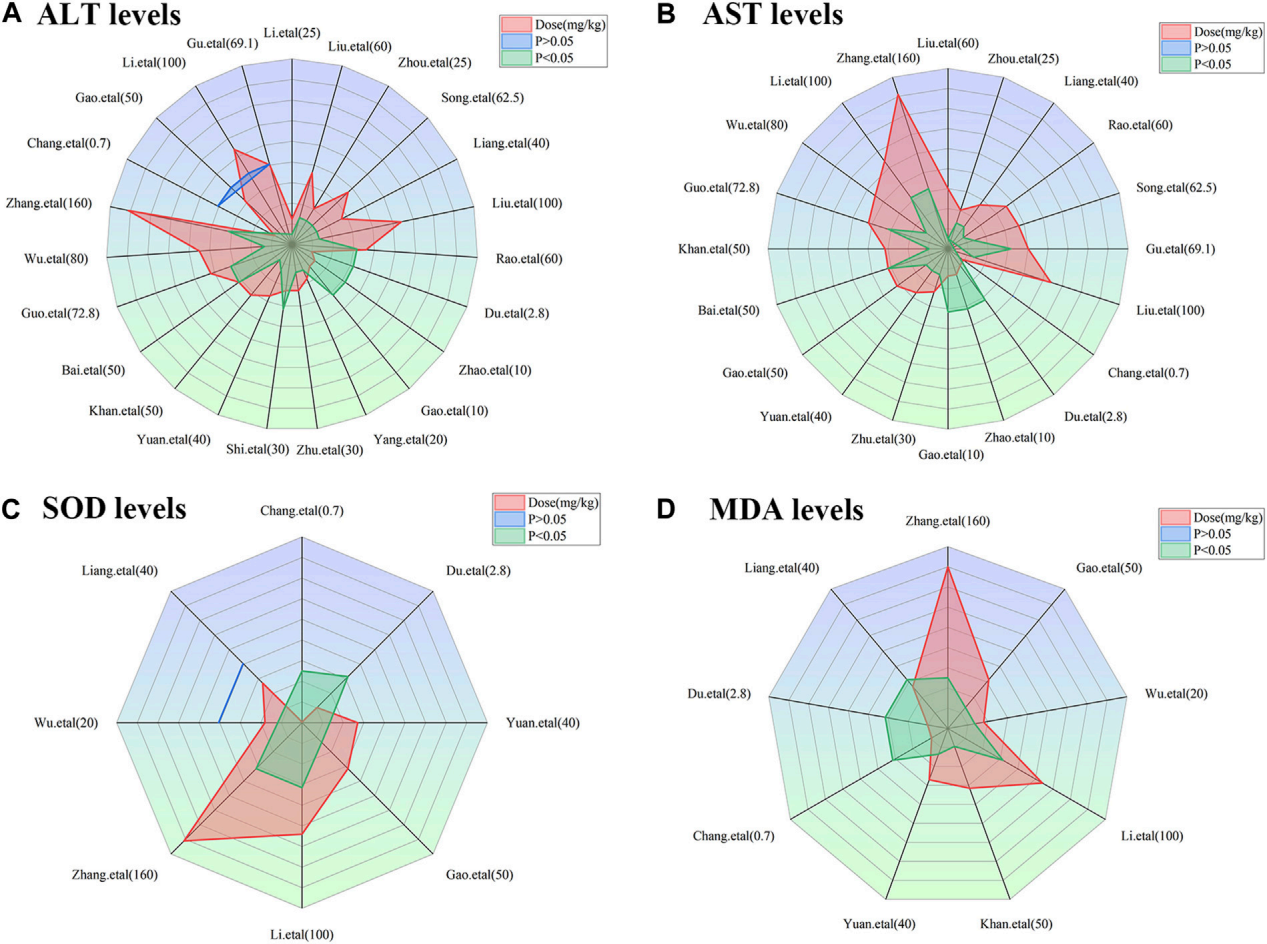
Radar charts of dose–time–effect relationship. **(A)** ALT levels; **(B)** AST levels; **(C)** SOD levels; **(D)** MDA levels.

To achieve effective treatment for a disease, it is crucial to not only use the appropriate medications but also carefully consider the dosage and duration of drug administration. The three key elements in clinical treatment are identifying the most suitable medication, determining the ideal dosage, and establishing the optimal timing. In this study, we utilized three-dimensional mappings and radar charts to analyze the treatment duration and dosage in each research, aiming to identify the optimal length of treatment and dosage for MT that would yield the most effective results. Figures 7, 8 displayed 3D maps and radar charts corresponding to the four key indications.

#### 3.6.1 The dose–time–effect/dose–time–toxicity relationship of ALT and AST levels

##### 3.6.1.1 Effective dose and time length of MT on ALT and AST levels

The ALT and AST levels can be effectively reduced by MT in LI models at a dose of 1.4 mg/kg/d to 100 mg/kg/d, if all other conditions (except the dose of MT) are suitable. However, if the MT dose is less than 1.4 mg/kg/d or greater than 100 mg/kg/d, these effects are not observed. To ascertain the precise MT dosage that is effective, additional research are necessary. Considering the treatment period, 3D maps and radar charts indicate that MT effectively decrease ALT and AST levels at 0.02 W-4 W, but is unsuccessful in reducing these levels over 4.29 W. However, if the treatment period was 0.02W–0.86W at a medium dose (25–50 mg/kg/d), MT reduced ALT and AST levels more effectively than at a low dose (0–25 mg/kg/d) or a high dose (>50 mg/kg/d). Further study needs to be performed to determine the specific effective dose and administration of MT for a treatment duration of more than 4.29 W (Figures 7A,B; Figures 8A,B).

##### 3.6.1.2 Toxic dose and time length of MT on ALT and AST levels

The levels of ALT and AST are increase by MT in normal models at a dosage of 10 mg/kg/d to 69.1 mg/kg/d, if all other conditions (except the dose of MT) are suitable. However, if the MT dose is less than 10 mg/kg/d or greater than 100 mg/kg/d, the toxicity of MT is not observed. Considering the treatment period, 3D maps and radar charts indicate that MT effectively increase ALT and AST levels at 1W-2 W or 8.57W-12.86W, but is unknown at 2W-8.57W. Additional investigation needs to be conducted to study the specific toxic dose and administration of MT *in vivo* (Figures 7A,B; Figures 8A,B).

#### 3.6.2 The impact of the effective dose and time length on SOD and MDA levels

According to the 3D maps and radar charts, the SOD levels in the MT groups were higher than those in the LI model groups at a dose of 1.4 mg/kg/d to 100 mg/kg/d. At 1W-4.29W, MT was found to increase the amounts of SOD in the MT groups. MT, in contrast with MDA, can lower MDA levels at a dosage of 0.7 mg/kg/d to 100 mg/kg/d and a duration of 0.14 W–4.29 W (Figures 7C,D; Figures 8C,D).

### 3.7 Potential mechanisms of action of MT

The hypothesized bilateral impacts of MT on LI are extensive and complicated. The identified signalling transduction pathways, namely, SERCA, SREBP1c/SCAP, Notch/RBP-J/HES1, IκK/NF-κB, Cul3/Rbx1/Keap1/Nrf2, and Bcl-2/ROS/Bax/caspase-9/caspase-3 have been evaluated in Supplementary Tables S5–S6.

### 3.8 Molecular docking of key targets

**FIGURE 9:**
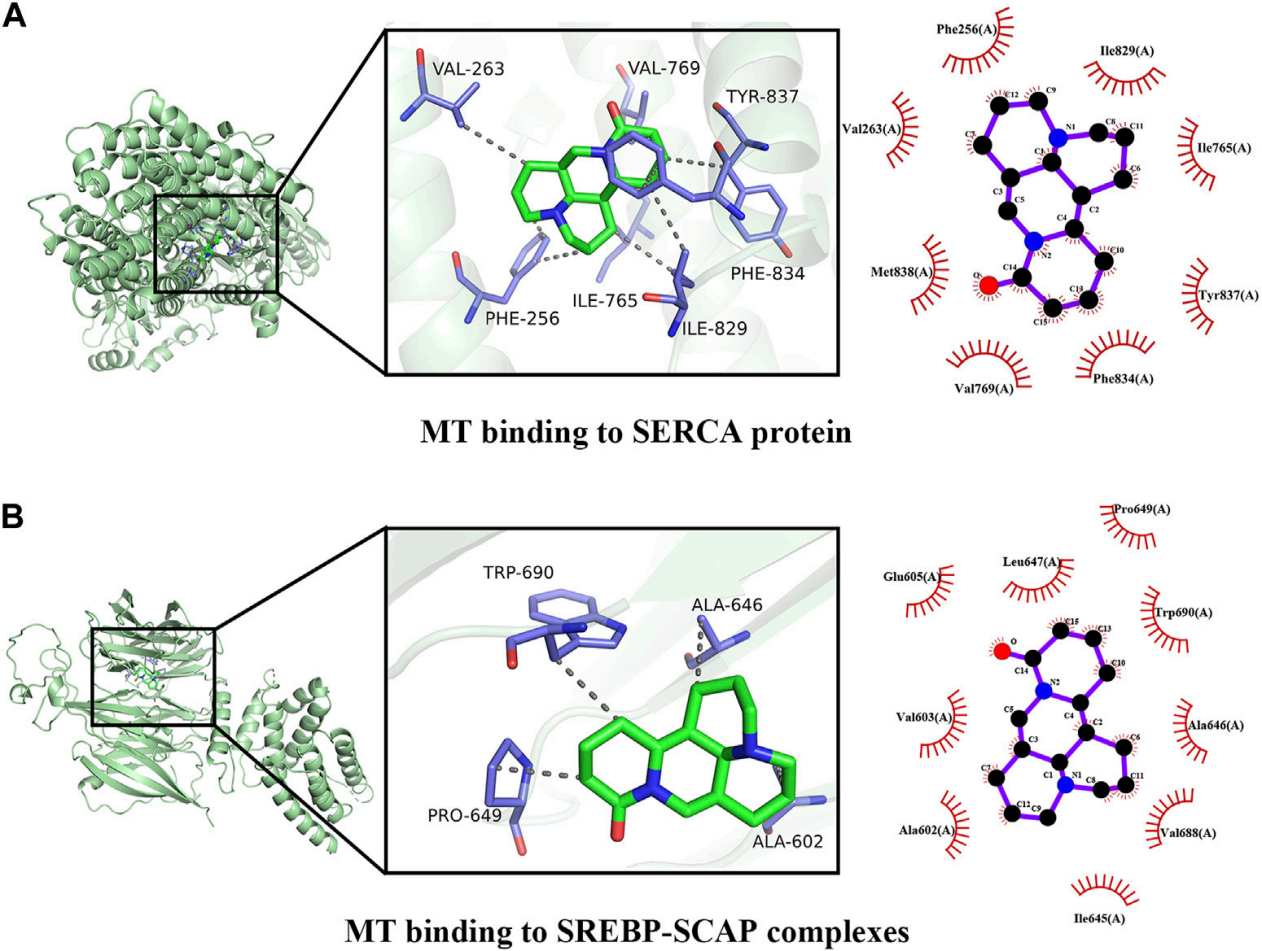
Docking patterns of MT and key targets. **(A)** MT binding to SERCA protein; **(B)** MT binding to SREBP-SCAP complexes.

To validate the potential mechanisms of action of MT, we utilized molecular docking to assess the binding affinity between MT and key targets. The molecular docking analysis demonstrated the interaction of MT with SERCA and SREBP-SCAP complexes, and the thermodynamic data was analyzed. The estimated free energy of −7.8 kcal/mol suggests that MT interacts with Phe256, Phe834, Ile829, Ile765, Tyr837, Val769, Val263, and Met83 on the SERCA protein. Additionally, with an estimated free energy of −6.8 kcal/mol, MT exhibits significant interactions with Glu605, Leu647, Pro649, Trp690, Ala646, Ala602, Val688, Val603, and Ile645 on the SREBP-SCAP complexes. These interactions between MT and the targets involve beneficial patterns of hydrogen bonds and hydrophobic interactions. The compound–target interactions were visualized using PyMoL 2.6 and Discovery Studio 2019 (Figure 9).

## 4 Discussion

**FIGURE 10:**
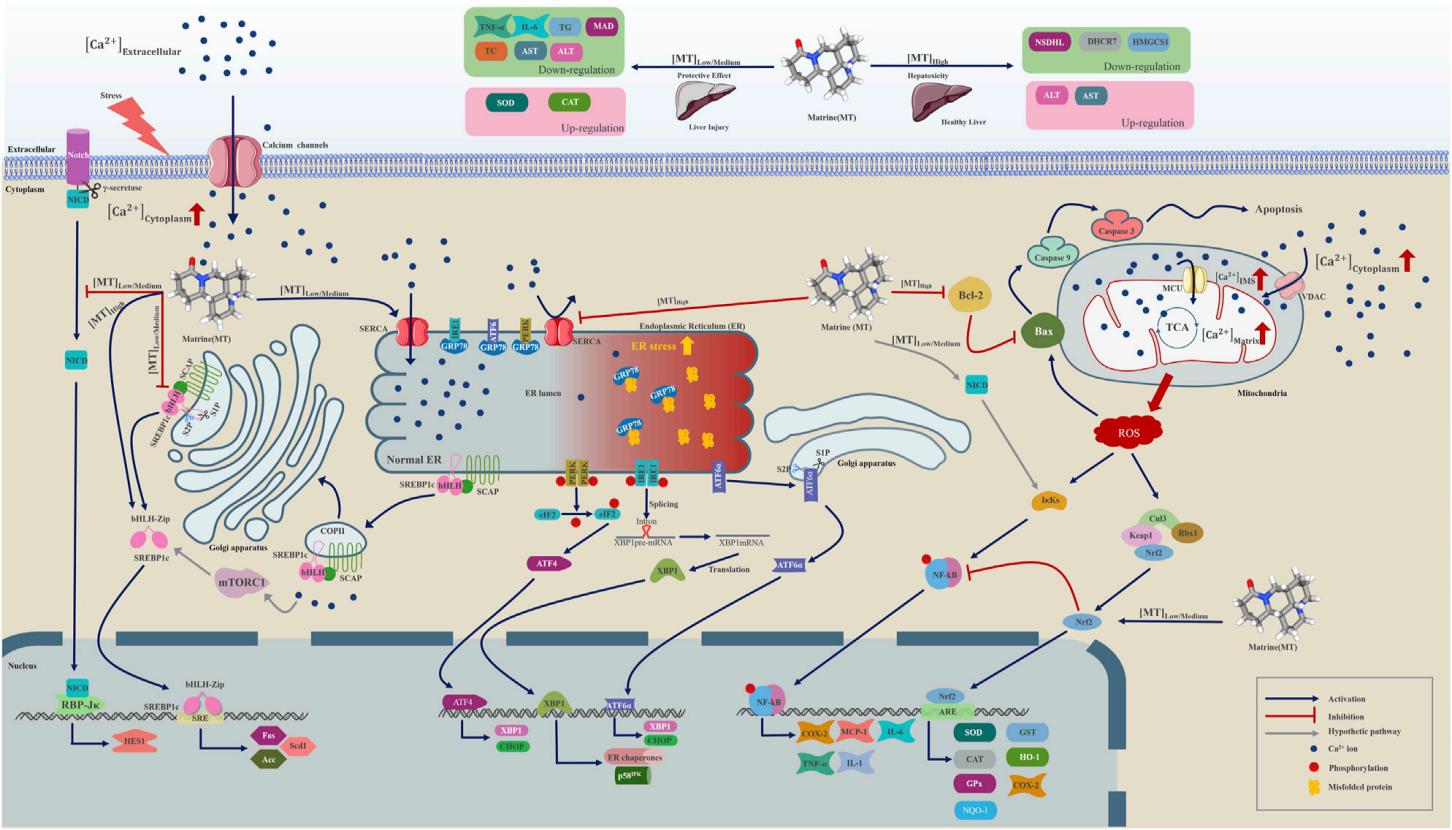
A graphical representation illustrates multiple molecular processes of matrine protection and toxicity in liver injury by modifying SERCA, SREBP1c/SCAP, Notch/RBP-J/HES1, IK/NF-B, Cul3/Rbx1/Keap1/Nrf2, and Bcl-2//Bax/caspase 9/caspase 3 signalling pathways. Matrine probably plays a significant role in Ca2+ homeostasis regulation within the endoplasmic reticulum, Golgi apparatus, and mitochondria. Matrine could additionally influence the expression of three essential metabolically related genes, including DHCR7, NSDHL, and HMGCS1. Please check the abbreviation list.

According to our meta-analysis, consisting of 24 published studies with 657 rodent models, MT provides information on liver protection and hepatotoxicity. We analyzed a range of indicators, such as TNF-α, IL-6, serum TG, serum TC, SOD, MDA, CAT, ALT, and AST, to establish the biological efficacy and diverse dosages of MT for treating and managing LI. Furthermore, by utilizing molecular docking techniques, we confirmed the interaction of MT with SERCA and SREBP-SCAP complexes, while also summarizing the mechanisms of MT as described in relevant literature. These findings aimed to gain a better understanding of the potential protective and harmful signaling pathways linked to the included indicators of MT on the LI (Figure 10).

### 4.1 The protective molecular mechanism of MT on LI

The comprehensive meta-analyses indicated that MT can protect from hepatotoxicity in animal models, and this protective effect is associated with variations in TNF-α, IL-6, serum TG, serum TC, MDA, ALT, AST, SOD, and CAT. Several signal transduction pathways are responsible for MT-induced alterations in these important indications of LI.

#### 4.1.1 MT could inhibit SREBP1c in the LI models

Sterol regulatory element-binding protein-1c (SREBP1c), a transcription factor which is generated from ER, might have a critical function in the regulation of lipogenesis and be activated by different nutrient states in the liver (Sekiya et al., 2008). SREBPs and SREBP cleavage activating protein (SCAP) interact to form a complex on the endoplasmic reticulum membrane. When cells in mammals lack cholesterol, SREBP-SCAP complexes assemble complexes in coat protein II (COPII) vesicles, facilitating transportation from the ER to the Golgi apparatus. To release the bHLH-Zip domains, SREBPs would be proteolyzed at the Golgi by site-1 and site-2 proteases (S1P and S2P) (Horton et al., 2002; Shimano and Sato, 2017; Lee et al., 2020). The translocated bHLH domain of SREBP1c interacts with the sterol regulatory element (SRE) in the nucleus, regulating the transcription of downstream lipid homeostasis genes such as fatty acid synthase (Fas), acetyl-CoA carboxylase (Acc), and stearoyl-CoA desaturase-1 (Scd1). SREBP1c also enhances the synthesis and accumulation of triacylglycerol (TG) in hepatocytes (Eberle et al., 2004). Previous studies have shown that the mechanistic target of rapamycin complex 1 mediates the nucleocytoplasmic transport of SREBP-1 and SREBP-2 (Peterson et al., 2011). A few findings indicate SREBP1c/2 may interact with NF-κB to modulate inflammation and cholesterol stability (Fowler et al., 2022; Guo et al., 2017). NF-κB enhances SCAP protein expression and promotes the activity of the SCAP-SREBP complex, which causes an inflammatory response and the accumulation of cholesterol (Lee et al., 2020; Li et al., 2013). In an animal model of nonalcoholic fatty liver disease (NAFLD) and nonalcoholic steatohepatitis (NASH) induced by a high-fat diet (HFD) or methionine-choline deficit (MCD), SREBP1c, Fas, Acc, ALT, AST, TNF-α, IL-6, and serum TC/TG levels are elevated. However, MT can reduce the levels of SREBP1c, Fas, Acc, ALT, AST, TNF-α, and IL-6 in hepatocytes of HFD and MCD mice by decreasing SREBP1c expression (Rinella et al., 2008; Gao et al., 2018). Modulation of SREBP1c may be positively correlated with the production of liver damage indicators, and MT may have a potential impact on hepatic injury biomarkers (Yang et al., 2022).

#### 4.1.2 MT might regulate Ca^2+^ homeostasis via SERCA to protect hepatocyte

Ca^2+^ is a second messenger that is required for cellular homeostasis through mTORC1, calmodulin, mitochondrial nitric oxide synthase, citric acid cycle (TCA cycle, Kreb cycle) and electron transport chain (ECT) and associated with ROS generation (Wan et al., 1989; O-Uchi et al., 2014; Jin et al., 2016; Tang et al., 2017; Diaz-Garcia et al., 2021; Stork et al., 2022). Extracellular stress stimuli such as CCL4, hepatic ischemia-reperfusion injury (HIRI), alcohol, and so on would increase Ca^2+^ transport from the extracellular area to the cytosol and mitochondria during the development of hepatic damage. The activation of Kupffer cells by hepatic I/R injuries is probably produced by the stimulation of store-operated Ca^2+^ channels (SOC), which increases Ca^2+^ influx into the cells and exacerbates the I/R-induced Kupffer cell injury (Pan et al., 2012). In rat liver, CCl4 might increase the expression and distribution of acid-sensing ion channel 1 (ASIC-1) (Pan et al., 2012). Consistent consumption of alcohol improves Ca^2+^-mediated mitochondrial permeability transition pore opening and raises cyclophilin D levels within the liver (King et al., 2010).

A steady Ca^2+^ concentration gradient across the cell membrane is maintained by eukaryotic cells (∼100 nM within the cytoplasm and ∼1 mM extracellular milieu) (Bagur and Hajnoczky, 2017). The ER and mitochondria play essential roles in the storage, transport, and upkeep of Ca2+ within the cell. Ca^2+^ dysregulation in ER and mitochondria is associated with LI, including chronic viral hepatitis, alcoholic liver disease, and nonalcoholic fatty liver disease (Li et al., 2007; Mantena et al., 2008; King et al., 2010; Xiao et al., 2017). The connection between ER stress and lipid metabolism is linked to intracellular Ca^2+^ homeostasis in the liver. Recent accumulating investigations have connected Ca^2+^ concentration disruption to ER stress, proving to be a significant risk factor during the progression of NAFLD to NASH, leading to increased inositol-requiring enzyme 1α(IRE1α), activating transcription factor 6α (ATF6α), phosphor-plasmic reticulum kinase (p-PERK), the 78 kDa glucose-regulated protein (GRP78), and C/EBP homologous protein (CHOP) expression (Bartlett et al., 2014; Park and Lee, 2014; Rieusset, 2017; Gao et al., 2018). Inactive GRP78, an ER chaperone protein, binds to three transmembrane unfolded protein response (UPR) stress sensors under physiological conditions: IRE1, ATF6α, and PERK. When unfolding proteins assemble within the ER lumen, raising ER stress, GRP78 dissociates from these UPRs to capture the unfolding proteins and activate the UPR stress sensors.

ATF6 is transported from the ER to the Golgi apparatus once it has been separated from GRP78, where it can be cleaved by S1P and S2P. An activated form of ATF6α might migrate to the nucleus and activate downstream target genes related to X-box-binding protein 1 (XBP1) and CHOP. ATF6α signaling pathways may be able to alleviate ER stress. A serine-threonine kinase domain and an endoribonuclease domain have been identified in IRE1. The active IRE1 endonuclease activity could remove the introns of XBP1 mRNA to generate spliced XBP1 (sXBP1) mRNA. The sXBP1 protein functions as a transcription factor, translocating into the nucleus to stimulate the production of ER chaperones and the HSP40 family member P58 ^IPK^ gene. Activated PERK might cause dimerization and autophosphorylation of the kinase, allowing it to phosphorylate eukaryotic initiation factor 2 (eIF2). Phosphorylated eIF2 could block new protein translation while minimizing ER stress, hence assisting cell survival via transcription factor 4 (ATF4) activation. ATF4 could cate to the nucleus and stimulates the production of the survival gene and the apoptotic cell death gene CHOP (Harding et al., 2003; Szegezdi et al., 2006).

Ca^2+^ is transported across the plasma membrane, the endoplasmic reticulum, and the mitochondria through Ca^2+^ channels. Prior studies have demonstrated that the sarco/endoplasmic reticulum Ca^2+^-ATPase (SERCA) pump, a member of the P-type ATPase family of ion channels, transports intracellular Ca^2+^ from the cytosol to the ER and maintains Ca^2+^ homeostasis between the cytoplasm and ER lumen. Diminished SERCA activity could increase cytosolic Ca^2+^ level, ER stress, and apoptosis in NALFD, whereas increased SERCA activity would reverse the process (Zhang et al., 2014; Lai et al., 2017). Lai et al. revealed that suppressing protein kinase C delta (PKCδ) could increase SERCA activity, thereby reducing ER stress (Lai et al., 2017). Meanwhile, Gao et al. demonstrated that exposing PA-induced L02 cells to low (200 μM) and medium (400 μM) doses of MT enhanced SERCA activity and facilitated Ca^2+^ influx from the cytosol into the ER lumen (Gao et al., 2018). These findings suggest that changes in SERCA function may contribute to the development of LI and provide a potential therapeutic target for various hepatic disorders.

#### 4.1.3 MT would downregulate Notch/RBP-J/HES1 signaling cascade in the LI

Previous research found that inhibiting Notch signaling using RBP-J deletion or a Notch inhibitor worsened hepatic I/R damage, demonstrated by impaired liver function and increased hepatocyte apoptosis (Yu H. C. et al., 2011; Yue et al., 2018). The intracellular transmembrane domain (NICD) of the Notch receptor is released through the catalytic action of an integral membrane protein γ-secretase complex. NICD might enter the nucleus and bind the DNA-binding protein RBP-J, allowing Notch target genes such as HES1 to be transcribed. Earlier research proposed that MT may diminish HES1 mRNA levels by downregulating RBP-Jκ mRNA expression to safeguard liver function and regeneration (Yang et al., 2013). The current study reveals that MT may promote hepatic progenitor cell development by obstructing the Notch/Jagged1/HES1 signaling pathway *in vivo* (Yang et al., 2016). By reducing SERCA activity and increasing ER stress in T-ALL cells, unmatured Notch signaling transduction pathways would be activated, contributing to apoptosis (De Ford et al., 2016). We hypothesize that MT can suppress the Notch/RBP-J/HES1 signaling cascade by promoting SERCA activity and minimize apoptosis in LI; however, more investigations are required.

#### 4.1.4 MT might indirectly regulate NF-κB activity via modifying SERCA in the LI

The nuclear factor kappa B (NF-κB) is a recognized transcription factor in pro-inflammatory pathways. NF-κB could potentially have an impact on controlling the processes of cell proliferation, differentiation, and cell death (Gerondakis et al., 2006; Khandelwal et al., 2011; Mitchell et al., 2016). TNF-α and IL-1, pro-inflammatory cytokines, may mediate NF-κB signaling transduction pathways and encourage downstream target gene expression. NF-κB activation will trigger the transcription and translation of COX-2, IL-6, MCP-1, TNF-α, and IL-1. The IκK complex, which is composed of α and β subunits, is necessary for NF-κB pathway activation via phosphorylation and ubiquitination. Previous literature has indicated that the activity of IκK and NF-κB is linked to various chronic liver injuries, including steatohepatitis, hepatocellular carcinoma, alcoholic liver disease, NAFLD, viral hepatitis, and biliary liver disease. Present results demonstrated that IκKα would interact with NICD directly to maintain the nuclear factor-kappa B (NF-κB) activity in the T-ALL cells model (Vacca et al., 2006), and we proposed that MT therapeutic effect on Ca^2+^ ion channel SERCA might regulate the process of inflammation and apoptosis in the LI through NICD/NF-κB interaction.

#### 4.1.5 MT could increase Nrf2 translocation to nucleus and protective effect

Under normal state, nuclear factor erythroid 2-related factor 2 (Nrf2), as a protective molecule, could have a crucial function in preventing oxidation in the liver. In a physiological condition, Nrf2 might attach to the kelch-like ECH-associated protein 1 (Keap1), which is an adaptor to the E3 ubiquitin ligase complex Cullin3 (Cul3)/ring box protein 1 (Rbx1), and subsequently be ubiquitinated and suppressed by Cul3 in the cytoplasm. Recently research showed oxidative stress, the main pathologic feature of most liver diseases, could modify Keap1 and inhibit Nrf2 ubiquitination (Kobayashi et al., 2006; Saito et al., 2016). Nrf2 accumulation in the cytoplasm would translocate into the nucleus and bind to antioxidant-responsive elements (ARE) to transcript anti-oxidative and anti-inflammation genes expression involving superoxide dismutase (SOD), glutathione-S transferase (GST), glutathione peroxidase (GPx), catalase (CAT), heme oxygenase-1 (HO-1), quinone oxidoreductase-1 (NQO-1), and cyclooxygenase-2 (COX-2) (Prestera et al., 1995; Raghunath et al., 2018; Bardallo et al., 2022). Additionally, active Nrf2 may inhibit NF-κBp65 phosphorylation and reduce NF-κBp65 translocation to the nucleus in animal models to minimize inflammation and apoptosis. The PERK may collaborate with Nrf2 to improve cell survival after exposure to ER stress (Cullinan et al., 2003; Dai et al., 2018). We identified that MT could enhance the protective impact against hepatic damage via Nrf2 moving to the nucleus and activate downstream transcription of genes including CAT, SOD, and HO-1 in HFD-induced liver injury mouse models (Zhang et al., 2013).

### 4.2 The hepatoxicity and molecular mechanism of MT on liver

As with the ER, mitochondria can potentially play an essential role in regulating Ca^2+^ homeostasis under physiological conditions. Specifically, Ca^2+^ could be transported to the outer membrane of mitochondria (OMM) through the voltage-dependent anion channels (VDACs) in the hepatocytes, and VDCAs would be regulated by a series of proteins, including inositol 1,4,5-trisphosphate receptors (IP3Rs), ryanodine receptor (RyR), glucose-regulated protein 75 (GRP75), and sigma-1 receptor (S1R), to transfer Ca^2+^ into the intermembranous space (IMS). Elevated Ca^2+^ levels in the IMS could lead the mitochondrial Ca^2+^ uniporter (MCU) on the inner mitochondrial membrane (IMM) to interact with the mitochondrial Ca^2+^ uptake 1/2 (MICU1/2) and promote Ca^2+^ influx to the matrix (Hirata et al., 2002; Csordas et al., 2013; Williams et al., 2015; Shoshan-Barmatz et al., 2018). Furthermore, previous research has suggested that matrix Ca^2+^ can influence the cycle of TCA and the process of oxidative phosphorylation for ATP production. Interestingly, the required ROS might be created simultaneously in aerobic metabolism to maintain microdomain cell signaling (Bertero and Maack, 2018).

Gao et al. observed that excessive amounts of MT (800 μM) elevated SREBP1c, Fas, and Acc expression in PA-induced L02 cells. In comparison to low and medium dosages of MT, overdosage treatment results in the opposite effect. High-MT treatment causes toxicity and ultimately loss of protective capacity in the PA-induced L02 cell line (Gao et al., 2018). Furthermore, at low and medium levels, MT might have a therapeutic function of active SERCA to increase Ca^2+^ ion influx to the ER in response to stress, but excessive MT would have a negative influence on this reaction. MT given in high doses inhibits SERCA activity, limiting Ca^2+^ transport from the cytosol to the ER lumen and increasing ER stress. Nonetheless, increased cytosolic Ca^2+^ may be transported across the mitochondria via VDACs, leading to a surge in Ca^2+^ accumulation within the IMS (Rapizzi et al., 2002; Shoshan-Barmatz et al., 2018). Ca^2+^ accumulation in the IMS stimulates the MCU to transport Ca^2+^ into the matrix. This process accelerates the metabolic rate of the TCA cycle and oxidative phosphorylation, resulting in an increase in mitochondrial ROS and apoptosis (Traaseth et al., 2004; Mallilankaraman et al., 2012; Csordas et al., 2013).

Overdosage of MT induces hepatotoxicity in animal models, and high-level MT may promote hepatocytes to produce higher ROS, increased HO-1, and the pro-apoptotic protein BAX while inhibiting the anti-apoptotic protein Bcl-2 synthesis. According to current research, mitochondrial ROS would activate NF-κB, improve the production of inflammatory cytokines, and prevent Nrf2 degradation, hence increasing HO-1 expression (Wang et al., 2015; Lingappan, 2018; Kasai et al., 2020; Liu et al., 2020; Li et al., 2021). The present literature has demonstrated that raising cytosolic Ca^2+^ concentration could also activate the NF-κB through elevating Ca2+/Calmodulin-Dependent Protein Kinase II (CaMKII) activity to phosphorylate and degrade IκK in the neurons (Snow and Albensi, 2016). Additionally, Rao et al. have discovered that MT-induced hepatotoxicity in the mice model suppresses three genes connected to steroid synthesis and metabolic processes in LI. These genes include 7-dehydrocholesterol reductase (DHCR7), NAD-(P)-dependent steroid dehydrogenase-like (NSDHL), and 3-hydroxy-3-methylglutaryl-coenzyme A synthase 1 (HMGCS1). However, the detailed mechanism is still to be further researched in the future (Rao et al., 2022).

### 4.3 The dual effects of matrine depend on dosage and molecular docking

The distinctive and dose-dependent effects of MT have been utilized to investigate various mechanisms of liver protection and hepatotoxicity. The suggested dosage, according to our data, is between 30–62. 5 mg/kg/d, which can be harmful to rodent animal models. When given at a dosage of 20 mg/kg/d from 0.02W to 0.86W, MT demonstrated significant liver protection with no hepatotoxicity. Finally, our findings show that a dosage of 20–30 mg/kg/d of 0.02–0.86 W has a considerable liver-protective effect with low hepatotoxicity. A dosage of more than 30–62.5 mg/kg/d of MT therapy, on the other hand, caused liver damage in animal models. Through the activation of SERCA, SREBP-SCAP complexes, and MT, the pharmacological actions of MT can produce both liver protection and damage. These complexes are responsible for linking the IRE1, ATF6, and PERK proteins, all of which play important roles in regulating ER stress. The interaction of MT with SERCA and SREBP-SCAP complexes was demonstrated utilizing molecular docking, and the thermodynamic data was analyzed. With an estimated free energy of −7.8 kcal/mol, the molecule MT interacts with Phe256, Phe834, Ile829, Ile765, Tyr837, Val769, Val263, and Met83 on the SERCA protein. With an estimated free energy of −6.8 kcal/mol, MT interacts substantially with the Glu605, Leu647, Pro649, Trp690, Ala646, Ala602, Val688, Val603, and Ile645 on the SREBP-SCAP complexes. SERCA and SREBP-SCAP complexes exhibit beneficial patterns of hydrogen bond and hydrophobic interactions.

### 4.4 Limitations

This meta-analysis adhered to the PRISMA standards (http://prisma-statement.org/), despite several limitations. 1) As only four English and four Chinese databases were utilized for article inclusion, selective bias was inevitable. In addition, we have not been able to compile all the relevant literature. 2) The heterogeneity of various studies could not be unified because of instrument index measurement error, different units of indicators, different experimental methods, *etc.* 3) Even though articles with quality scores of less than 5 points were disregarded, there may be heterogeneity in the results due to differences in the quality of the included articles. 4) The absence of a standardized method for animal intervention, drug dosage, treatment regimens, and model species across studies was another factor that might have caused the high heterogeneity. The reliability of MT’s results in treating LI or causing hepatotoxicity was confirmed by the sensitivity analysis, Egger’s test, and subgroup analysis. 5) Although the primary pharmacological mechanisms of MT in terms of liver protection and hepatotoxicity have been summarized, not all mechanisms could be summarized due to the complex pathogenic mechanisms involved. 6) The reliability of MT for hepatotoxicity may be lower than for hepatoprotection because only 5 articles on this condition were included. Future research should be conducted to investigate the hepatotoxicity of MT. 7) For ethical reasons, there is a paucity of literature on the toxicological effects of MT in the human body. Therefore, we only included studies using animal models. It is important to conduct relevant clinical trials to confirm the efficacy and reliability of MT in the clinical management of hepatoprotection and hepatotoxicity. 8) We validated the binding of MT to key proteins using molecular docking, but experiments were still needed to prove it.

Although this meta-analysis has several limitations, the findings may provide new strategies for clinical medication and drug development.

## 5 Conclusion

In summary, our study revealed that within the dose range of 10–69.1 mg/kg and time range of 1–2 weeks, MT could have a bilateral impact on liver damage. However, at a dose of 20–30 mg/kg/d for 0.02–0.86 weeks, it demonstrated high protection and low toxicity on the liver. Molecular docking analysis indicated that MT interacts with SERCA and SREBP-SCAP complexes. These interactions involve beneficial patterns of hydrogen bonds and hydrophobic interactions. By activating SERCA, a Ca^2+^ ion channel on the ER, MT could play a crucial role in regulating Ca^2+^ homeostasis in damaged hepatocytes. This helps maintain the balance among the cytoplasm, ER, Golgi apparatus, and mitochondria. Our findings suggest that MT doses ranging from 1.4 mg/kg/d to 100 mg/kg/d may have a preventive and therapeutic effect on LI by modulating the expression of biomarkers such as TNF-α, IL-6, serum TG, serum TC, SOD, MDA, CAT, ALT, and AST. Additionally, signaling pathways such as SREBP1c/SCAP, Notch/RBP-J/HES1, IκK/NF-κB, and Cul3/Rbx1/Keap1/Nrf2 are likely involved in the protective process. It is interesting to note that many of these signaling pathways directly or indirectly interact with Ca^2+^ homeostasis. However, in normal hepatocytes, a high dosage of MT can suppress SERCA activity, leading to an adverse impact on Ca^2+^ homeostasis. This, in turn, can cause hepatotoxicity and promote apoptosis through the reduction of Bcl-2 and activation of the Ros/Bax/caspase 9/caspase 3 pathway. Elevated MT levels can also modulate the expression of various metabolic indicators, including AST, ALT, DHCR7, NSDHL, and HMGCS1. Further investigation is required to fully understand how MT influences the expression of these genes.

## Figures and Tables

**Table:** 

**Acc**	Acetyl-coenzyme A-carboxylase
**ALT**	Alanine aminotransferase
**ARE**	Antioxidant-responsive elements
**AST**	Aspartate aminotransferase
**ASIC-1**	Acid-sensing ion channel 1
**ATF4**	Transcription factor 4
**ATF6α**	Activating transcription factor 6α
**Bax**	Bcl-2-associated X protein
**Bcl-2**	B-cell lymphoma-2
**C**	Control
**CAT**	Catalase
**Ca** ^**2+**^	Calcium ion
**CaMKII**	Ca2+/Calmodulin-Dependent Protein Kinase II
**CHOP**	C/EBP homologous protein
**Cul3**	Cullin3
**COPII**	Complexes in coat protein II
**COX-2**	Cyclooxygenase 2
**DHCR7**	7-dehydrocholesterol reductase
**DILI**	Drug-induced liver injury
**EASL**	European Association for the Study of the Liver
**ECT**	Electron transport chain
**ER**	Endoplasmic reticulum
**eIF2**	eukaryotic initiation factor 2
**Fas**	Fatty acid synthase
**GRP75**	Glucose-regulated protein 75
**GRP78**	78 kDa glucose-regulated protein
**GST**	Glutathione-S transferase
**GPx**	Glutathione peroxidase
**H**	High
**HES1**	Hairy and enhancer of split 1
**HFD**	High-fat die
**HIRI**	Hepatic ischemia-reperfusion injury
**HMGCS1**	3-hydroxy-3-methylglutaryl-coenzyme A synthase 1
**HO-1**	Heme oxygenase-1
**I**	Intervention
**I** ^**2**^	I-squared
**ICR mice**	Institute of Cancer Research mice
**IL-1**	Interleukin 1
**IL-6**	Interleukin 6
**IRE1α**	Inositol-requiring enzyme 1 α
**IMS**	Intermembranous space
**IMM**	Inner mitochondrial membrane
**IP3Rs**	Inositol 1,4,5-trisphosphate receptors
**Keap1**	kelch-like ECH-associated protein 1
**L**	Low
**M**	Medium
**MCD**	Methionine choline deficient
**MCU**	Mitochondrial Ca^2+^ uniporter
**MDA**	Malondialdehyde
**MICU1/2**	Mitochondrial Ca^2+^ uptake 1/2
**MT**	Matrine
**mTORC1**	mechanistic target of rapamycin complex 1
**NASH**	Nonalcoholic steatohepatitis
**NAFLD**	Nonalcoholic fatty liver disease
**NAFLD**	Non-alcoholic fatty liver disease
**NICD**	Intracellular transmembrane domain
**Nrf2**	Nuclear factor erythroid 2-related factor 2
**NSDHL**	NAD-(P)-dependent steroid dehydrogenase-like
**NPS**	Natural products
**NF-κB**	Nuclear factor kappa-B
**NQO-1**	Quinone oxidoreductase-1
**OMM**	Outer membrane of mitochondria
**PKCδ**	Protein kinase C delta
**p-PERK**	phosphor-plasmic reticulum kinase
**Rbx1**	Ring box protein 1
**RyR**	Ryanodine receptor
**ROS**	Reactive oxygen species
**RBP-J(κ)**	Recombination signal binding protein (kappa) J
**Scd1**	Stearoyl-coenzyme A desaturase 1
**SCAP**	SREBP cleavage activating protein
**SD**	Standard deviation
**SERCA**	Sarco/endoplasmic reticulum Ca^2+^-ATPase
**SOD**	Superoxide dismutase
**SOC**	Store-operated Ca^2+^ channels
**SRE**	Sterol regulatory element
**SREBP**	Sterol regulatory element binding protein
**sXBP1**	spliced XBP1
**S1P**	Site-1 proteases
**S2P**	Site-2 proteases
**S1R**	Sigma-1 receptor
**TC**	Total cholesterol
**TCM**	Traditional Chinese medicine
**TG**	Triglyceride
**TGF-β**	Transforming growth factor beta
**TNF-α**	Tumor necrosis factor alpha
**UPR**	Unfolded protein response
**VDACs**	Voltage-dependent anion channels
**W**	Week(s)
**WHO**	World Health Organization
**XBP1**	X-box-binding protein 1
**95%CI**	95% confidence interval

## Data Availability

The original contributions presented in the study are included in the article/Supplementary Material, further inquiries can be directed to the corresponding authors.
