# Supplementary material for: The dynamic equilibrium between the protective and toxic effects of matrine in the development of liver injury: a systematic review and meta-analysis
Source: Front Pharmacol. 2024 Jan 29;15:1315584. doi: 10.3389/fphar.2024.1315584 (PMC10859759; doi:10.3389/fphar.2024.1315584)
Supplement: Supplementary file 1 [file DataSheet1.docx]

| ***Supplementary table 1: Retrieval strategy*** | |
| --- | --- |
| **Number** | **Search terms** |
| #1 | Matrine[MeSH]  Matrine[Title/Abstract] OR MT[Title/Abstract] OR Matrines OR (1R,2R,9S,17S)-7,13-diazatetracyclo[7.7.1.02,7.013,17]heptadecan-6-one [Title/Abstract] OR C_15_H_24_N_2_O OR Matrine Alkaloids OR Matrine, (5beta)-Isomer) OR Matrine-type Alkaloids OR Sophoridine |
| #2 | Liver[MeSH]  hepatic injury[Title/Abstract] OR Liver injury[Title/Abstract] OR Hepatoprotection[Title/Abstract] |
| #3 | #1 AND #2 |
| #4 | Liver[MeSH]  hepatic injury[Title/Abstract] OR Liver injury[Title/Abstract] OR hepatotoxicity[Title/Abstract] OR hepatic toxicity[Title/Abstract] OR Liver toxicity[Title/Abstract] |
| #5 | #1 AND #4 |
| #6 | #3 OR #5 |

| ***Supplementary Table 2: Stratified analysis of main outcome indicators from studies of hepatoprotection of MT*** | | | | | | |
| --- | --- | --- | --- | --- | --- | --- |
| Indicators | Experiments(n) | Individuals(n) | SMD | 95%CI | P-value | Heterogeneity |
| **ALT** | 36 | 532 | -3.42 | (-4.34,-2.50) | P<0.0001 | 90.3% |
| **AST** | 30 | 448 | -3.76 | (-4.74,-2.78) | P<0.0001 | 87.5% |
| **MDA** | 16 | 130 | -2.70 | (-3.59,-1.81) | P<0.0001 | 64.4% |
| **SOD** | 14 | 120 | +4.00 | (2.66,5.33) | P<0.0001 | 75.6% |
| **Serum TG** | 10 | 100 | -1.68 | (-2.70,-0.67) | 0.001 | 77.0% |
| **Serum TC** | 8 | 88 | -1.69 | (-2.59,-0.78) | P<0.0001 | 68.6% |
| **TNF-α** | 16 | 326 | -3.72 | (-5.50,-1.95) | P<0.0001 | 94.6% |
| **IF-6** | 10 | 254 | -3.77 | (-4.87,-2.67) | P<0.0001 | 66.8% |
| **CAT** | 8 | 72 | +2.93 | (2.23,3.62) | P<0.0001 | 0.0% |

| ***Supplementary Table 3: Stratified analysis of subgroups of hepatoprotection of MT*** | | | | | | |
| --- | --- | --- | --- | --- | --- | --- |
| Indicators | Subgroups | Individuals(n) | SMD | 95%CI | P-value | Heterogeneity |
| **ALT** | Rats | 394 | -3.70 | (-4.96,-2.44) | P<0.0001 | 92.2% |
|  | Mice | 138 | -3.05 | (-4.42,-1.67) | P<0.0001 | 85.9% |
|  | H | 256 | -2.33 | (-3.85,-0.81) | 0.003 | 92.3% |
|  | M | 112 | -5.88 | (-8.90,-2.86) | P<0.0001 | 93.6% |
|  | L | 164 | -3.05 | (-4.32,-1.77) | P<0.0001 | 85.8% |
|  | ≥4W | 96 | -1.14 | (-1.77,-0.51) | P<0.0001 | 47.5% |
|  | <4W | 436 | -4.38 | (-5.47,-3.29) | P<0.0001 | *88.0%* |
| **AST** | Rats | 346 | -3.68 | (-4.75,-2.60) | P<0.0001 | *85.8%* |
|  | Mice | 102 | -4.50 | (-6.84,-2.16) | P<0.0001 | *89.8%* |
|  | H | 256 | -2.52 | (-3.52,-1.51) | P<0.0001 | *80.7%* |
|  | M | 96 | -6.18 | (-9.62,-2.74) | P<0.0001 | *91.3%* |
|  | L | 96 | -4.51 | (-6.93,-2.08) | P<0.0001 | *90.6%* |
|  | ≥4W | 80 | -1.60 | (-2.32,-0.88) | P<0.0001 | *45.3%* |
|  | <4W | 368 | -4.71 | (-5.89,-3.53) | P<0.0001 | *83.5%* |
| **SOD** | Rats | 72 | 5.41 | （3.30,7.51） | P<0.0001 | *73.7%* |
|  | Mice | 48 | 2.58 | （1.79,3.38） | P<0.0001 | *0.0%* |
|  | H | 56 | 3.75 | (1.57,5.93) | 0.001 | *81.8%* |
|  | M | 36 | 6.70 | (2.85,10.54) | 0.001 | *67.4%* |
|  | L | 28 | 2.74 | (1.66,3.82) | P<0.0001 | *0.0%* |
|  | ≥4W | 60 | 4.73 | (2.77,6.70) | P<0.0001 | *71.7%* |
|  | <4W | 60 | 3.32 | (1.72,4.92) | P<0.0001 | *70.6%* |
| **MDA** | Rats | 72 | -2.47 | (-3.10,-1.84) | P<0.0001 | *0.0%* |
|  | Mice | 58 | -3.52 | (-5.82,-1.23) | 0.003 | *83.6%* |
|  | H | 56 | -2.35 | (-3.05,-1.65) | P<0.0001 | *0.0%* |
|  | M | 46 | -4.02 | (-2.55,-0.02) | 0.006 | *81.5%* |
|  | L | 28 | -4.51 | (-5.66,0.24) | 0.071 | *85.4%* |
|  | ≥4W | 60 | -2.45 | (-3.14,-1.76) | P<0.0001 | *0.0%* |
|  | <4W | 70 | -3.12 | (-4.82,-1.42) | P<0.0001 | *78.2%* |

| ***Supplementary Table 4: Stratified analysis of main outcome indicators from studies of hepatotoxicity of MT*** | | | | | | |
| --- | --- | --- | --- | --- | --- | --- |
| Indicators | Experiments(n) | Individuals(n) | SMD | 95%CI | P-value | Heterogeneity |
| ALT | 10 | 105 | +1.91 | （0.79，3.02） | P<0.0001 | 81.3% |
| AST | 10 | 105 | +2.21 | （0.75，3.68） | P<0.0001 | 87.9% |

| ***Supplementary Table 5: Mechanisms of included studies of matrine in the hepatotoxicity*** | | | |
| --- | --- | --- | --- |
| ***Category*** | ***Studies*** | ***Year*** | ***Proposed mechanisms*** |
| ***Toxicity*** | ***Song.etal*** | ***2009*** | Matrine increase the WBC, PLT, GR, AST and ALT levels, which may cause inflammation or local infection in the body, causing liver damage. |
| ***Toxicity*** | ***Liang.etal*** | ***2015*** | Matrine causes hepatotoxicity by activating CYP450 and producing excessive amounts of harmful active substances such as free radicals, electrophilic radicals, and oxygen radicals.  Matrine induces hepatocyte apoptosis by promoting the binding of death receptors Fas-R, TNF-R1, and TRAIL to death ligands. |
| ***Toxicity*** | ***Gu.etal*** | ***2019*** | Matrine promotes hepatocellular apoptosis, necrosis or aberrant proliferation by activating CYP450 enzymes (notably cytochrome P450 2B (CYP2B)). |
| ***Toxicity*** | ***Liu.etal*** | ***2020*** | Matrine inhibits ROS generation in hepatocytes and causes mitochondrial dysfunction. Matrine causes hepatotoxicity by increasing oxidative stress and inflammation through the Nrf2-mediated HO-1 signaling pathway. |
| ***Toxicity*** | ***Rao.etal*** | ***2022*** | Matrine causes hepatotoxicity by several metabolic pathways (riboflavin metabolism, purine metabolism, and ascorbate and aldarate metabolism). |

| ***Supplementary Table 6: Mechanisms of included studies of matrine in the hepatoprotection*** | | | |
| --- | --- | --- | --- |
| ***Category*** | ***Studies*** | ***Year*** | ***Proposed mechanisms*** |
| ***Protection*** | ***Li.etal*** | **2005** | Matrine can inhibit Con A-induced LI by inhibiting T cell activation, IFN-γ levels and TNF-α levels. |
| ***Protection*** | ***Liu.etal*** | ***2008*** | Matrine protects rats against HIRI-induced acute LI and alleviates inflammatory injury of hepatic sinusoidal endothelial cells by inhibiting expression of IL-6 and TNF-α. |
| ***Protection*** | ***Zhou.etal*** | ***2009*** | Matrine can inhibit the expression of Fas and FasL in liver tissue of mice with ConA-induced LI, thereby reducing hepatocyte apoptosis and exerting an anti-liver injury effect. |
| ***Protection*** | ***Yang.etal*** | ***2013*** | Matrine protects rats against LI through downregulation of the Notch-RBP-Jκ signaling pathway and its effects on specific signaling pathways that implement this mechanism. |
| ***Protection*** | ***Shi.etal*** | ***2013*** | Matrine have an protective effect on the hepatic infiltration of the inflammatory Gr1hi monocyte subset in injured livers, which is most possibly through its inhibition of both MCP-1 production and activity. |
| ***Protection*** | ***Zhang.etal*** | ***2013*** | Matrine protects rats against progression from HFD-induced LI to nonalcoholic steatohepatitis through the inhibition of lipid peroxidation, revision of oxidative status, diminished proinflflammatory cytokines and insulin resistance. And activation of the Nrf2 signal pathway seems to be related to this process. |
| ***Protection*** | ***Gao.etal*** | ***2013*** | Low-dose matrine has a protective effect on alcoholic liver injury, and its mechanism is related to inhibiting oxidative stress, reducing lipid peroxidation, and regulating lipid metabolism. |
| ***Protection*** | ***Tang.etal*** | ***2013*** | Matrine can inhibit the mRNA transcription and protein expression levels of COX-2 and iNOS in the liver of HFD model rats, which indicated can treat HFD-induced LI by inhibiting oxidative stress and inflammation. |
| ***Protection*** | ***Wu.etal*** | ***2014*** | Matrine can protect mice against acute alcoholic liver injury through increasing the activities of SOD and GSH in liver tissue, reducing ROS in liver cells, and reducing the content of transaminases in serum. |
| ***Protection*** | ***Zhu.etal*** | ***2015*** | Matrine protects mice against HIRI-induced liver injury through inhibition of activating Kupffer cell and TNF-α levels. |
| ***Protection*** | ***Zhao.etal*** | ***2015*** | Matrine protects mice CCl_4_-induced liver injury through antiinflammatory (ROS, TNF-α, IL-6). |
| ***Protection*** | ***Li.etal*** | ***2016*** | Matrine can enhance the antioxidant capacity of rats with chronic alcoholic liver injury and has the effect of inhibiting lipid peroxidation. |
| ***Protection*** | ***Guo.etal*** | ***2017*** | Matrine can inhibit the expression of CYP2E1 protein and protect mitochondria to treat CCl_4_-induced liver injury. |
| ***Protection*** | ***Gao.etal*** | ***2018*** | As a competitive inhibitor of SERCA, matrine is able to improve the state of ER stress, and then alleviates lipid metabolic disorder, mitochondrial dysfunction and infammatory reaction, all of which participate in non-alcoholic fatty liver disease pathological progression. |
| ***Protection*** | ***Bai.etal*** | ***2018*** | Matrine inhibits hepatocyte apoptosis by inhibiting the TRAIL/BAX/caspase-3 pathway. |
| ***Protection*** | ***Khan.etal*** | ***2019*** | Matrine has significant antidepressant and anxiolytic effects in acute liver injury models through modulation of CCl4-induced neuroinflammation, oxidative stress, reduced neurogenesis and apoptosis. |
| ***Protection*** | ***Yuan.etal*** | ***2020*** | Matrine protects mice against HIRI-induced liver injury through inhibiting MKK7/JNK pathway and p38 phosphorylation. |
| ***Protection*** | ***Chang.etal*** | ***2021*** | Matrine protects mice APAP-induced liver injury through antiinflammatory, antioxidation and anti-apoptosis. |
| ***Protection*** | ***Du.etal*** | ***2021*** | Matrine protects mice against acute alcoholic liver injury through antioxidation and eliminating radical. |


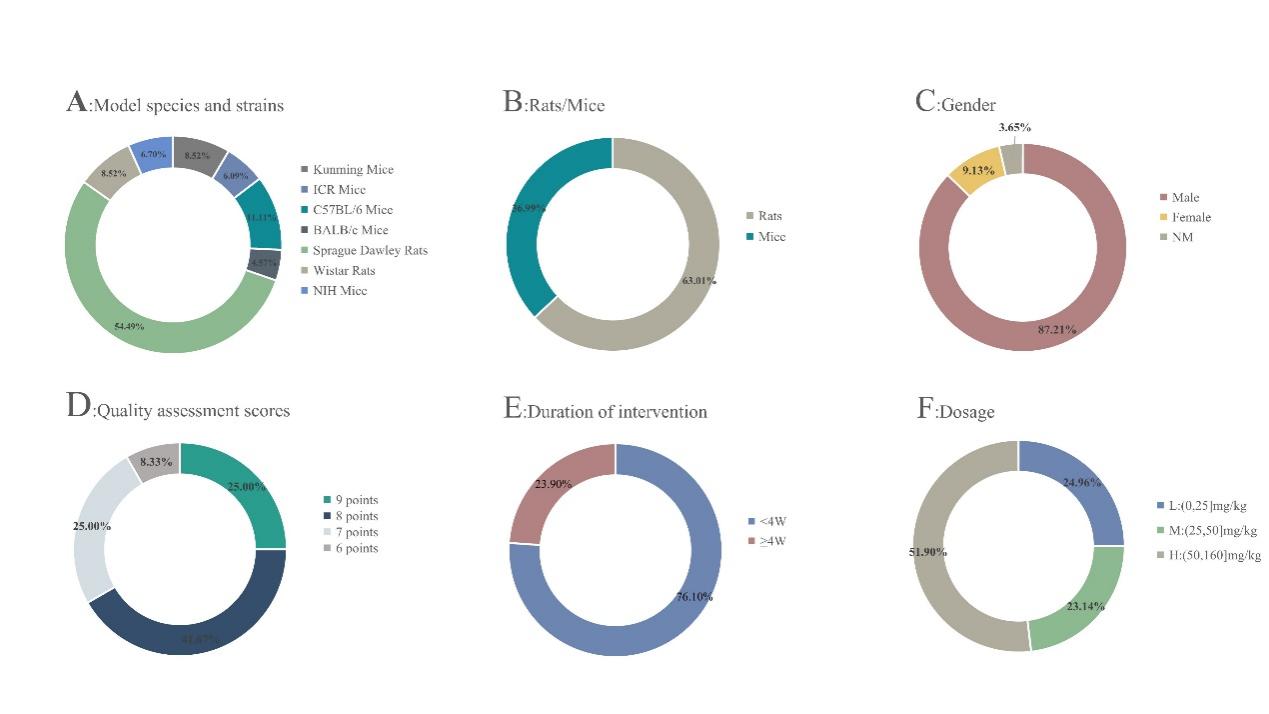


**Supplementary figure 1** Characteristics of eligible studies. (A) Model species and strains; (B) Rats/mice; (C)Gender; (D) Quality assessment scores; (E) Duration of intervention;(F) Dosage.


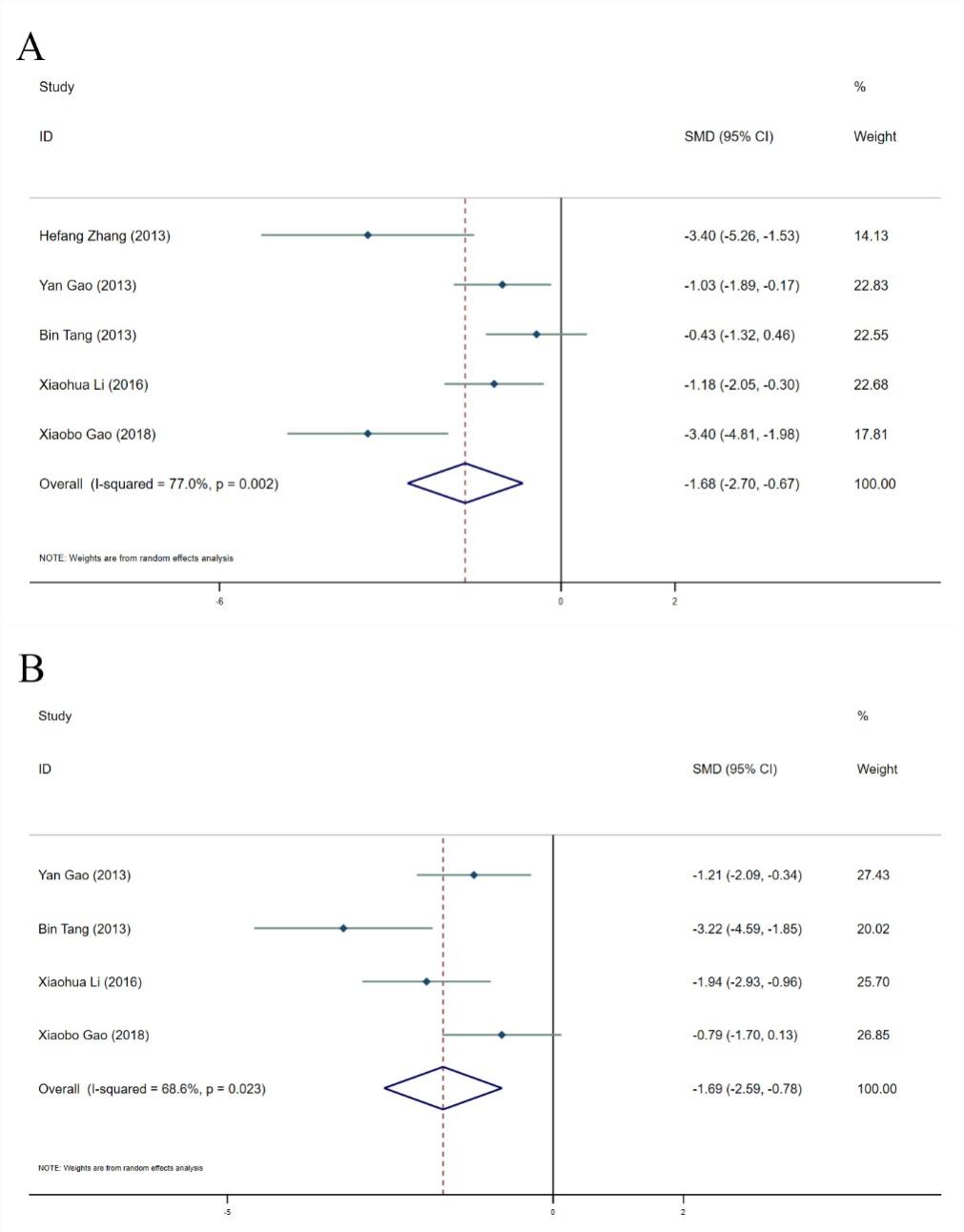


**Supplementary figure 2** Forest plot (effect size and 95% CI) summarising the effects of MT on hepatoprotection. (A) Serum TG levels; (B) Serum TC levels.


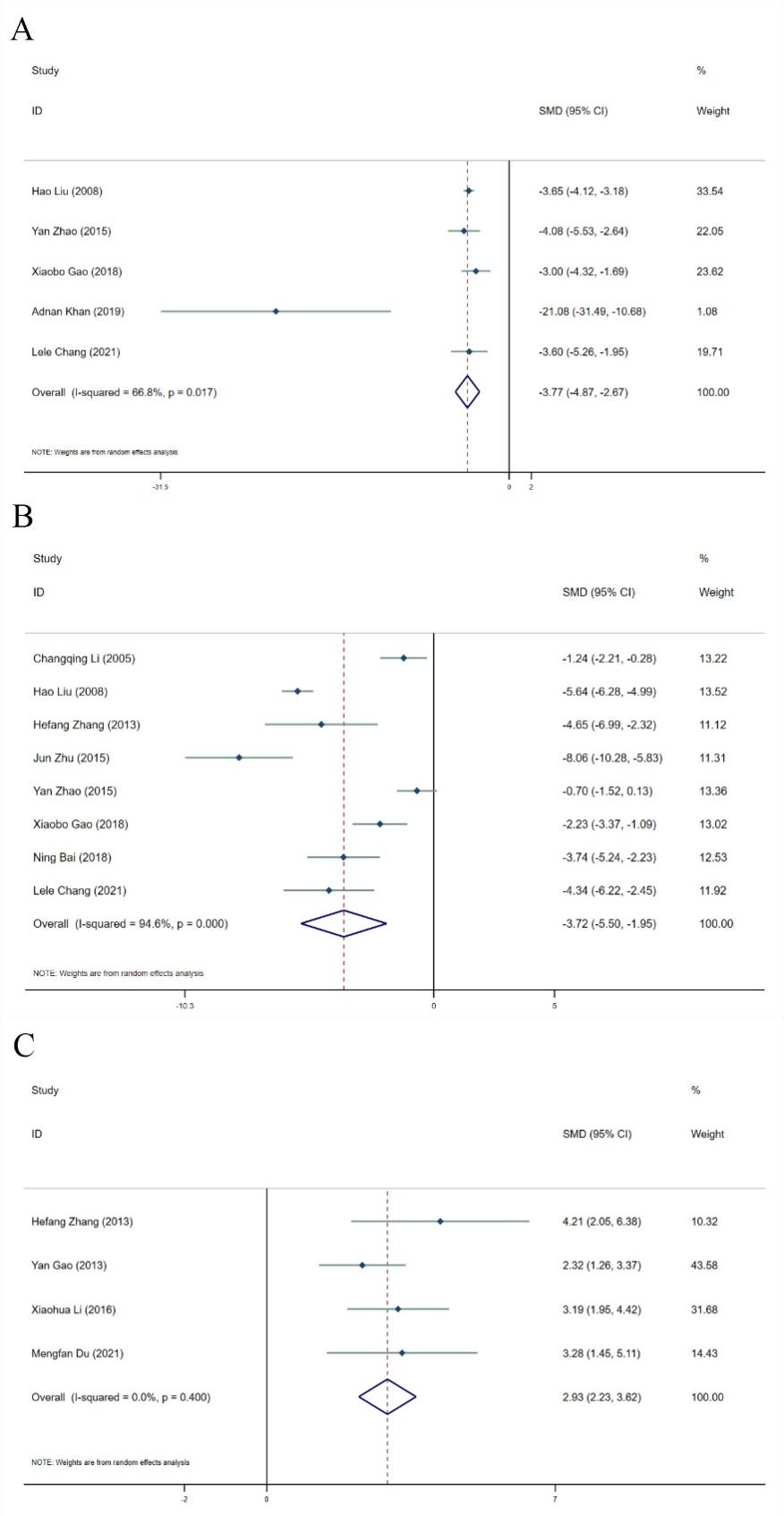


**Supplementary figure 3** Forest plot (effect size and 95% CI) summarising the effects of MT on hepatoprotection. (A) IL-6 levels; (B)TNF levels; (C)CAT levels.


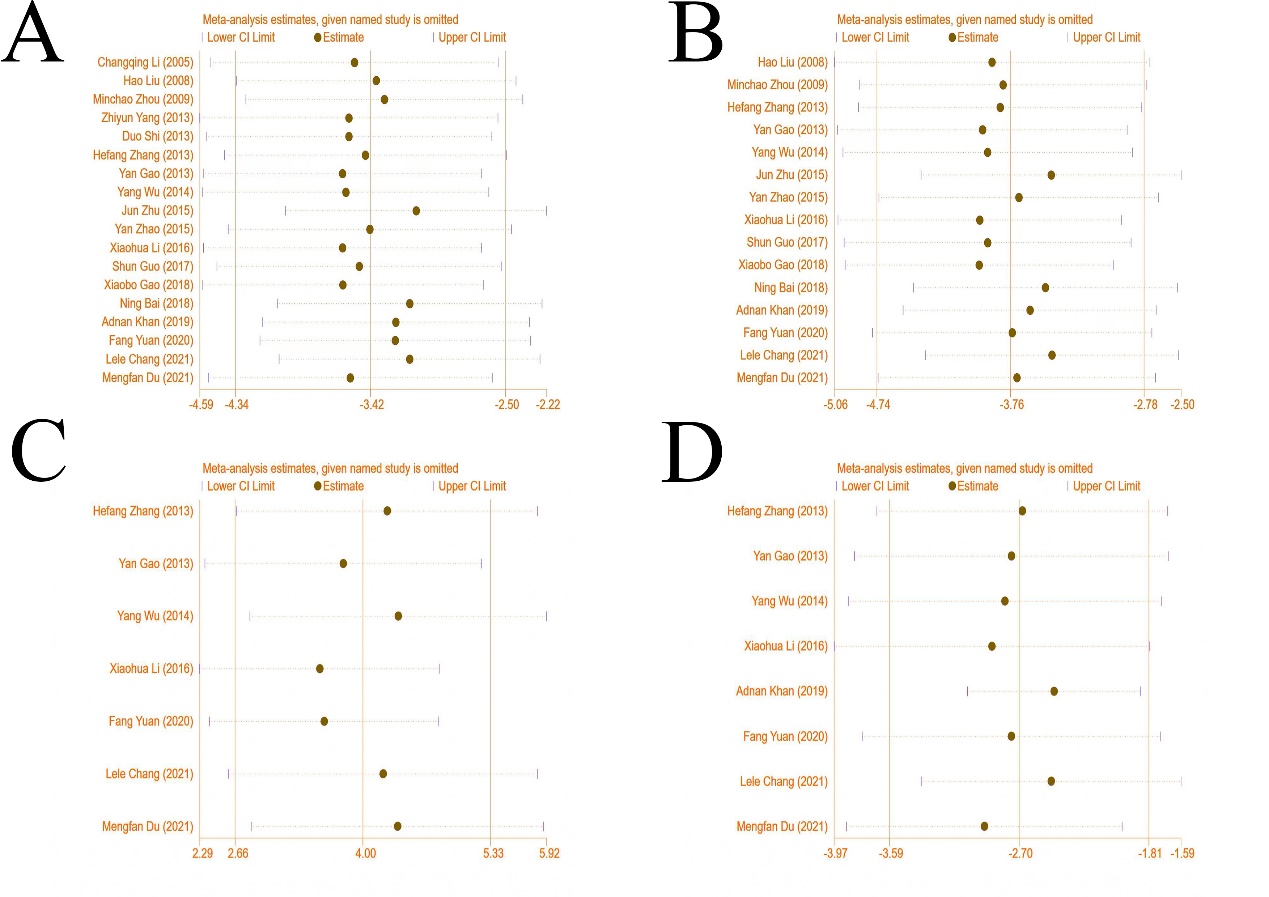


**Supplementary figure 4** The plot of Sensitivity test in hepatoprotection. (A)ALT; (B)AST; (C)SOD; (D)MDA.


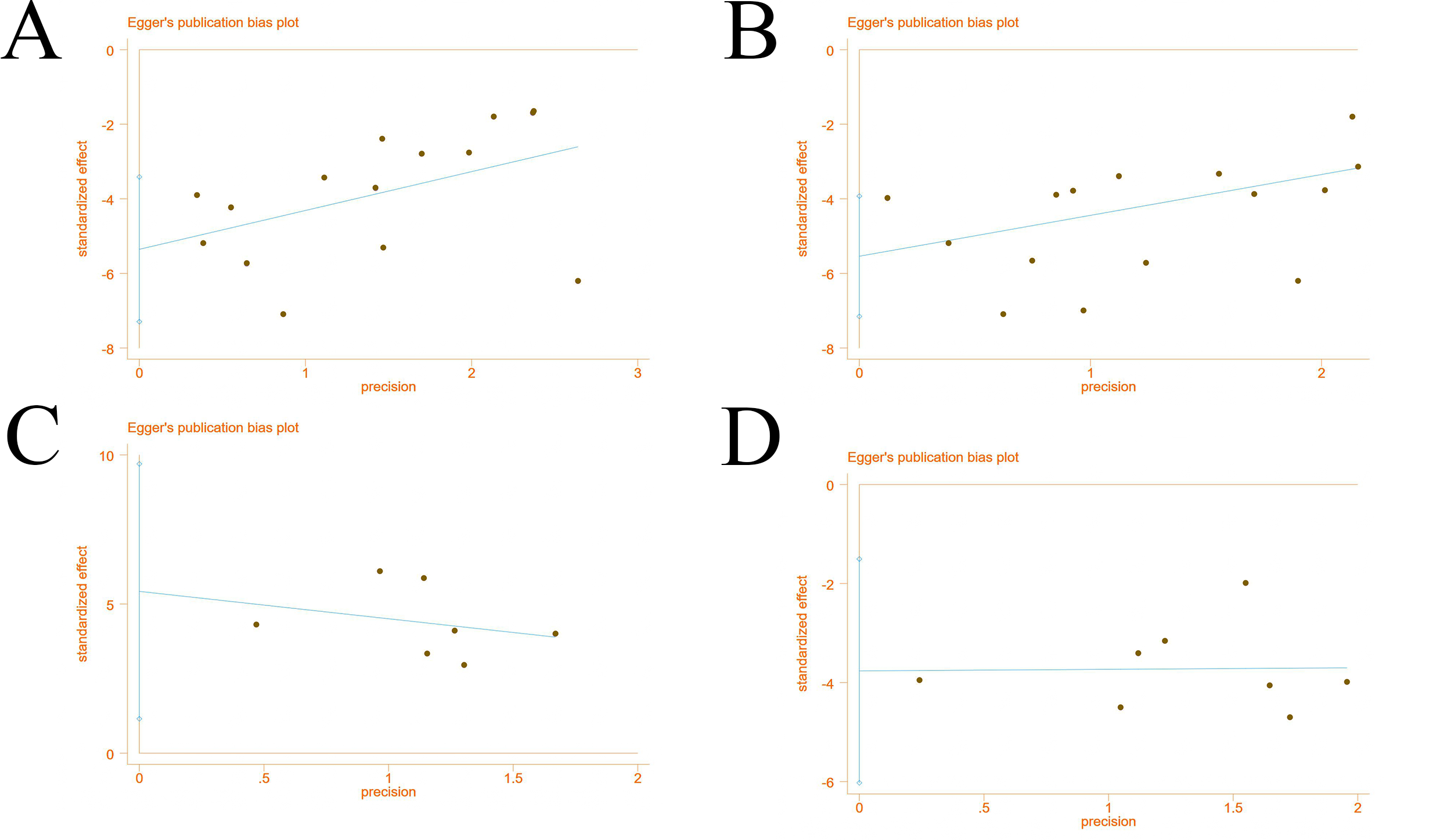


**Supplementary figure 5** Egger’s publication bias plot in hepatoprotection. (A)ALT, |t| = |-1.79|; (B)AST, |t| = |-1.92|; (C)SOD, |t| = |3.26|; (D)MDA, |t| = |-4.08|)

| **Section and Topic** | **Item #** | **Checklist item** | **Location where item is reported** |
| --- | --- | --- | --- |
| **TITLE** | | |  |
| Title | 1 | Identify the report as a systematic review. | √ |
| **ABSTRACT** | | |  |
| Abstract | 2 | See the PRISMA 2020 for Abstracts checklist. | √ |
| **INTRODUCTION** | | |  |
| Rationale | 3 | Describe the rationale for the review in the context of existing knowledge. | √ |
| Objectives | 4 | Provide an explicit statement of the objective(s) or question(s) the review addresses. | √ |
| **METHODS** | | |  |
| Eligibility criteria | 5 | Specify the inclusion and exclusion criteria for the review and how studies were grouped for the syntheses. | √ |
| Information sources | 6 | Specify all databases, registers, websites, organisations, reference lists and other sources searched or consulted to identify studies. Specify the date when each source was last searched or consulted. | √ |
| Search strategy | 7 | Present the full search strategies for all databases, registers and websites, including any filters and limits used. | √ |
| Selection process | 8 | Specify the methods used to decide whether a study met the inclusion criteria of the review, including how many reviewers screened each record and each report retrieved, whether they worked independently, and if applicable, details of automation tools used in the process. | √ |
| Data collection process | 9 | Specify the methods used to collect data from reports, including how many reviewers collected data from each report, whether they worked independently, any processes for obtaining or confirming data from study investigators, and if applicable, details of automation tools used in the process. | √ |
| Data items | 10a | List and define all outcomes for which data were sought. Specify whether all results that were compatible with each outcome domain in each study were sought (e.g. for all measures, time points, analyses), and if not, the methods used to decide which results to collect. | √ |
|  | 10b | List and define all other variables for which data were sought (e.g. participant and intervention characteristics, funding sources). Describe any assumptions made about any missing or unclear information. | √ |
| Study risk of bias assessment | 11 | Specify the methods used to assess risk of bias in the included studies, including details of the tool(s) used, how many reviewers assessed each study and whether they worked independently, and if applicable, details of automation tools used in the process. | √ |
| Effect measures | 12 | Specify for each outcome the effect measure(s) (e.g. risk ratio, mean difference) used in the synthesis or presentation of results. | √ |
| Synthesis methods | 13a | Describe the processes used to decide which studies were eligible for each synthesis (e.g. tabulating the study intervention characteristics and comparing against the planned groups for each synthesis (item #5)). | √ |
|  | 13b | Describe any methods required to prepare the data for presentation or synthesis, such as handling of missing summary statistics, or data conversions. | √ |
|  | 13c | Describe any methods used to tabulate or visually display results of individual studies and syntheses. | √ |
|  | 13d | Describe any methods used to synthesize results and provide a rationale for the choice(s). If meta-analysis was performed, describe the model(s), method(s) to identify the presence and extent of statistical heterogeneity, and software package(s) used. | √ |
|  | 13e | Describe any methods used to explore possible causes of heterogeneity among study results (e.g. subgroup analysis, meta-regression). | √ |
|  | 13f | Describe any sensitivity analyses conducted to assess robustness of the synthesized results. | √ |
| Reporting bias assessment | 14 | Describe any methods used to assess risk of bias due to missing results in a synthesis (arising from reporting biases). | √ |
| Certainty assessment | 15 | Describe any methods used to assess certainty (or confidence) in the body of evidence for an outcome. | √ |
| **RESULTS** | | |  |
| Study selection | 16a | Describe the results of the search and selection process, from the number of records identified in the search to the number of studies included in the review, ideally using a flow diagram. | √ |
|  | 16b | Cite studies that might appear to meet the inclusion criteria, but which were excluded, and explain why they were excluded. | √ |
| Study characteristics | 17 | Cite each included study and present its characteristics. | √ |
| Risk of bias in studies | 18 | Present assessments of risk of bias for each included study. | √ |
| Results of individual studies | 19 | For all outcomes, present, for each study: (a) summary statistics for each group (where appropriate) and (b) an effect estimate and its precision (e.g. confidence/credible interval), ideally using structured tables or plots. | √ |
| Results of syntheses | 20a | For each synthesis, briefly summarise the characteristics and risk of bias among contributing studies. | √ |
|  | 20b | Present results of all statistical syntheses conducted. If meta-analysis was done, present for each the summary estimate and its precision (e.g. confidence/credible interval) and measures of statistical heterogeneity. If comparing groups, describe the direction of the effect. | √ |
|  | 20c | Present results of all investigations of possible causes of heterogeneity among study results. | √ |
|  | 20d | Present results of all sensitivity analyses conducted to assess the robustness of the synthesized results. | √ |
| Reporting biases | 21 | Present assessments of risk of bias due to missing results (arising from reporting biases) for each synthesis assessed. | √ |
| Certainty of evidence | 22 | Present assessments of certainty (or confidence) in the body of evidence for each outcome assessed. | √ |
| **DISCUSSION** | | |  |
| Discussion | 23a | Provide a general interpretation of the results in the context of other evidence. | √ |
|  | 23b | Discuss any limitations of the evidence included in the review. | √ |
|  | 23c | Discuss any limitations of the review processes used. | √ |
|  | 23d | Discuss implications of the results for practice, policy, and future research. | √ |
| **OTHER INFORMATION** | | |  |
| Registration and protocol | 24a | Provide registration information for the review, including register name and registration number, or state that the review was not registered. | √ |
|  | 24b | Indicate where the review protocol can be accessed, or state that a protocol was not prepared. | √ |
|  | 24c | Describe and explain any amendments to information provided at registration or in the protocol. | √ |
| Support | 25 | Describe sources of financial or non-financial support for the review, and the role of the funders or sponsors in the review. | √ |
| Competing interests | 26 | Declare any competing interests of review authors. | √ |
| Availability of data, code and other materials | 27 | Report which of the following are publicly available and where they can be found: template data collection forms; data extracted from included studies; data used for all analyses; analytic code; any other materials used in the review. | √ |

*From:*  Page MJ, McKenzie JE, Bossuyt PM, Boutron I, Hoffmann TC, Mulrow CD, et al. The PRISMA 2020 statement: an updated guideline for reporting systematic reviews. BMJ 2021;372:n71. doi: 10.1136/bmj.n71

For more information, visit: <http://www.prisma-statement.org/>
